# Systematic review and meta-analysis of tumor biomarkers in predicting prognosis in esophageal cancer

**DOI:** 10.1186/1471-2407-13-539

**Published:** 2013-11-11

**Authors:** Meilan Chen, Jizheng Huang, Zhenli Zhu, Jun Zhang, Ke Li

**Affiliations:** 1 Department of Preventive Medicine, Shantou University Medical College, No.22 Xinling Road, Shantou, Guangdong 515041, China

**Keywords:** Esophageal cancer, Tumor biomarkers, Prognosis, Survival, Meta-analysis

## Abstract

**Background:**

Esophageal cancer (EC) is a frequently occurring cancer with poor prognosis despite combined therapeutic strategies. Many biomarkers have been proposed as predictors of adverse events. We sought to assess the prognostic value of biomarkers in predicting the overall survival of esophageal cancer and to help guide personalized cancer treatment to give patients the best chance at remission.

**Methods:**

We conducted a systematic review and meta-analysis of the published literature to summarize evidence for the discriminatory ability of prognostic biomarkers for esophageal cancer. Relevant literature was identified using the PubMed database on April 11, 2012, and conformed to the REMARK criteria. The primary endpoint was overall survival and data were synthesized with hazard ratios (HRs).

**Results:**

We included 109 studies, exploring 13 different biomarkers, which were subjected to quantitative meta-analysis. Promising markers that emerged for the prediction of overall survival in esophageal squamous cell cancer included VEGF (18 eligible studies, n = 1476, HR = 1.85, 95% CI, 1.55-2.21), cyclin D1 (12 eligible studies, n = 1476, HR = 1.82, 95% CI, 1.50-2.20), Ki-67 (3 eligible studies, n = 308, HR = 1.11, 95% CI, 0.70-1.78) and squamous cell carcinoma antigen (5 eligible studies, n = 700, HR = 1.28, 95% CI, 0.97-1.69); prognostic markers for esophageal adenocarcinoma included COX-2 (2 eligible studies, n = 235, HR = 3.06, 95% CI, 2.01-4.65) and HER-2 (3 eligible studies, n = 291, HR = 2.15, 95% CI, 1.39-3.33); prognostic markers for uncategorized ECs included p21 (9 eligible studies, n = 858, HR = 1.27, 95% CI, 0.75-2.16), p53 (31 eligible studies, n = 2851, HR = 1.34, 95% CI, 1.21-1.48), CRP (8 eligible studies, n = 1382, HR = 2.65, 95% CI, 1.64-4.27) and hemoglobin (5 eligible studies, n = 544, HR = 0.91, 95% CI, 0.83-1.00).

**Conclusions:**

Although some modest bias cannot be excluded, this review supports the involvement of biomarkers to be associated with EC overall survival.

## Background

Esophageal cancer (EC), which accounted for 482,300 new cases of cancer in 2008, is the eighth most common cancer worldwide, and has the sixth highest incidence of cancer mortality, with 406,800 deaths registered [1]. Although the prevalence is highest in Africa and Asia, the incidence of adenocarcinoma is rising in western countries and the America [2-4]. Surgery, combined with neoadjuvant radiation and chemotherapy, or even neoadjuvant chemoradiotherapy, remains the only curative modality for EC. However, the long-term prognosis of patients undergoing potentially curative esophageal resection is still poor, with the reported 5-year survival rate being 9.8% [5]. Commonly used classification systems utilize histological type to group EC into two main categories: esophageal squamous cell carcinoma (ESCC) and adenocarcinoma (EADC). ESCC can occur in all parts of the esophagus, whereas EADC arises mostly via metaplasia of the epithelium of the distal esophagus. Published studies of EC may not distinguish between ESCC and EADC.

The ability to predict patients with poor prognosis would help guide surgery and adjuvant treatment according to individual risk. Attempts have been made to predict poor prognosis in patients with EC using clinicopathological characteristics. Age, tumor stage distribution, tumor histology and body mass index have all been found to predict survival [6-8]. The ability to predict tumor behavior on the basis of molecular markers from either biopsy or serum samples would help inform the patients and clinician during the decision-making process. With advances in the understanding of tumor biology, there is sufficient new evidence available to gain further insight into this disease. In addition, biomarkers of prognostic significance, may present novel therapeutic targets.

The aim of this study was to summarize the results of published studies regarding the prognostic role of the molecular markers in EC. In this review, we prioritized the available data, in all included surveys, according to either the REMARK (Reporting recommendations for tumor MARKer prognostic studies) study design or methodological assessment quality metrics [9]. Many variations exist in the experimental methods chosen and procedures used, including antigen retrieval observed variability in staining pattern description, cut-off point selection, and assignment of specimens to categories, that influence the prognostic value of the proposed association. Because new biomarkers should enhance the current routine prognostic markers to be adopted for use in the clinic, studies that do not extend their statistical analysis beyond univariate survival measures are less valuable than studies that do. We sought to determine candidate biomarkers with sufficient evidence to support prospective validation in a controlled clinical environment and to identify functional pathways for which data either suggest a lack of involvement in EC prognosis or the need for additional investigation due to insufficient rigor among the previously conducted studies. We identified a subset of candidate predictors of EC outcome from the published literature that were evaluated according to robust sampling and laboratory methods.

## Methods

### Search strategy

To identify all primary research studies that evaluated levels of candidate biomarker expression as a prognostic factor among individuals with EC, we searched the PubMed medical literature database up to April 11, 2012, without language restrictions, using a strategy developed with an expert librarian based on terms for esophageal carcinoma, prognostic studies [10] and biomarkers. The search strategy was based on combinations of (“esophageal” or “esophagus”), (“neoplasms” or “carcinoma” or “tumor”), (gene or protein or biomarker or marker), and (prognos* or “survival analysis” [Mesh] or “follow-up studies” [Mesh] or mortality [Majr] or mortality[subheading] or incidence [Mesh] or predict or course or outcome). One reviewer (M. Chen) obtained the full texts of relevant articles following the search and inspection of titles and abstracts of citations to identify those articles that were likely to report the study of prognostic biomarkers in EC. In cases where data in several publications were derived from part or all of the same patient series, only the study presenting the most recent or most complete dataset was included.

### Methodology and validity assessment

We used published guidelines for reporting tumor marker studies and quality metrics for evaluating studies to include in the cancer-related meta-analyses [9,11]. Criteria used to determine study eligibility were as follows: 1) a prospective or retrospective cohort design with a well-defined study population and justification for all excluded eligible cases, 2) assay of the primary EC specimens, 3) a clear description of methods for specimen handling and testing, including selection and preparation of reagents or kits, as well as visualization techniques, 4) clear statements on the choice of positive/present and negative/absent controls and on assay validation, 5) statistical analysis using multivariable proportional hazards modeling that adjusted for clinical prognostic factors, and 6) reporting of the resultant adjusted hazard ratios (HRs) and their 95% confidence intervals (CIs), or provision of data available for statistical estimation of HRs. Because esophageal small cell carcinomas, epidermoid cancer of the esophagus, and neuroendocrine carcinoma of the esophagus have different clinical courses, studies that did not distinguish these tumor types from EC were excluded.

Quality assessment was performed in duplicated for each eligible study by two independent reviewers (Chen and Huang) using operationalized prognostic biomarker reporting guidelines [9] and extract details on 16 items (Additional file 1: Table S1). This scale allowed for assessment of study design, biomarker measurement, outcome and analysis.

### Data extraction

Two investigators (Chen and Zhu) reviewed all eligible studies and carefully extracted study characteristics in duplicate, including the first author’s name, publication year, country of origin, histology, sample size, gender, mean/median age, disease stage, test method, cutoff value, the status of biomarker expression, and the computed multivariable hazard ratio and its 95% CI. When results were present without confidence intervals, the p value was used to estimate the confidence intervals via the z-statistic.

### Statistical methods

All eligible individual biomarker assays were sorted according to their major biological function. Function was determined by reviewing the current scientific literature comprehensively and then classifying according to the 5 acquired capabilities of cancer as defined by Hanahan and Weinberg [12], and which included sustained angiogenesis, evasion of apoptosis, insensitivity to antigrowth signals, limitless replicative potential and tissue invasion and metastasis. To accommodate blood biomarkers, the Hanahan-Weinberg classification system was supplemented by one additional category: serum markers. Biomarkers evaluated in less than five studies, were excluded in this review and out of quantitative synthesis. For biomarkers assayed in five or more studies, the summary HR and 95% CI were calculated by using fixed effects according to generic inverse variance and random effects model using the DerSimonian-Laird method [13]. Statistical heterogeneity among studies was assessed using Q and I^2^ statistics [14]. We considered that heterogeneity was present when the Q-test P-value was less than 0.1. In addition, when I^2^ was lower than 50%, studies with an acceptable heterogeneity were considered, and the fixed-effects model was used; otherwise, a random effect model was adopted. The combined HRs were estimated graphically by Forest plots. Possible source of heterogeneity were investigated by subgroup analysis. Study publication bias was assessed with counter-enhanced funnel plots, by Begg’s adjusted rank correlation test and by Egger’s regression asymmetry test [15-17]. When p > 0.05 was considered to indicate that there was no publication bias in the studies. All statistical analysis was conducted using Stata SE 11.0 software (Stata Corporation).

## Results

### Eligible studies

**Figure 1:**
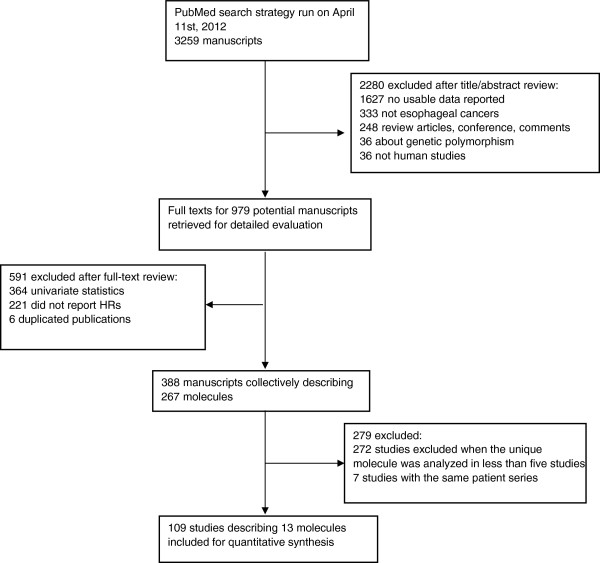
Flow diagram of the literature search and selection of eligible studies.

The abstracts and titles of 3259 primary manuscripts were identified for initial review using strategies as described. Reviewers identified 979 manuscripts to be appropriate in terms of evaluation of prognostic biomarkers in EC. For these manuscripts, full-text articles were obtained. Upon further review, 109 studies published between 1994 and 2012 were eligible for this systematic review and with meta-analysis (Figure 1).

**Figure 2:**
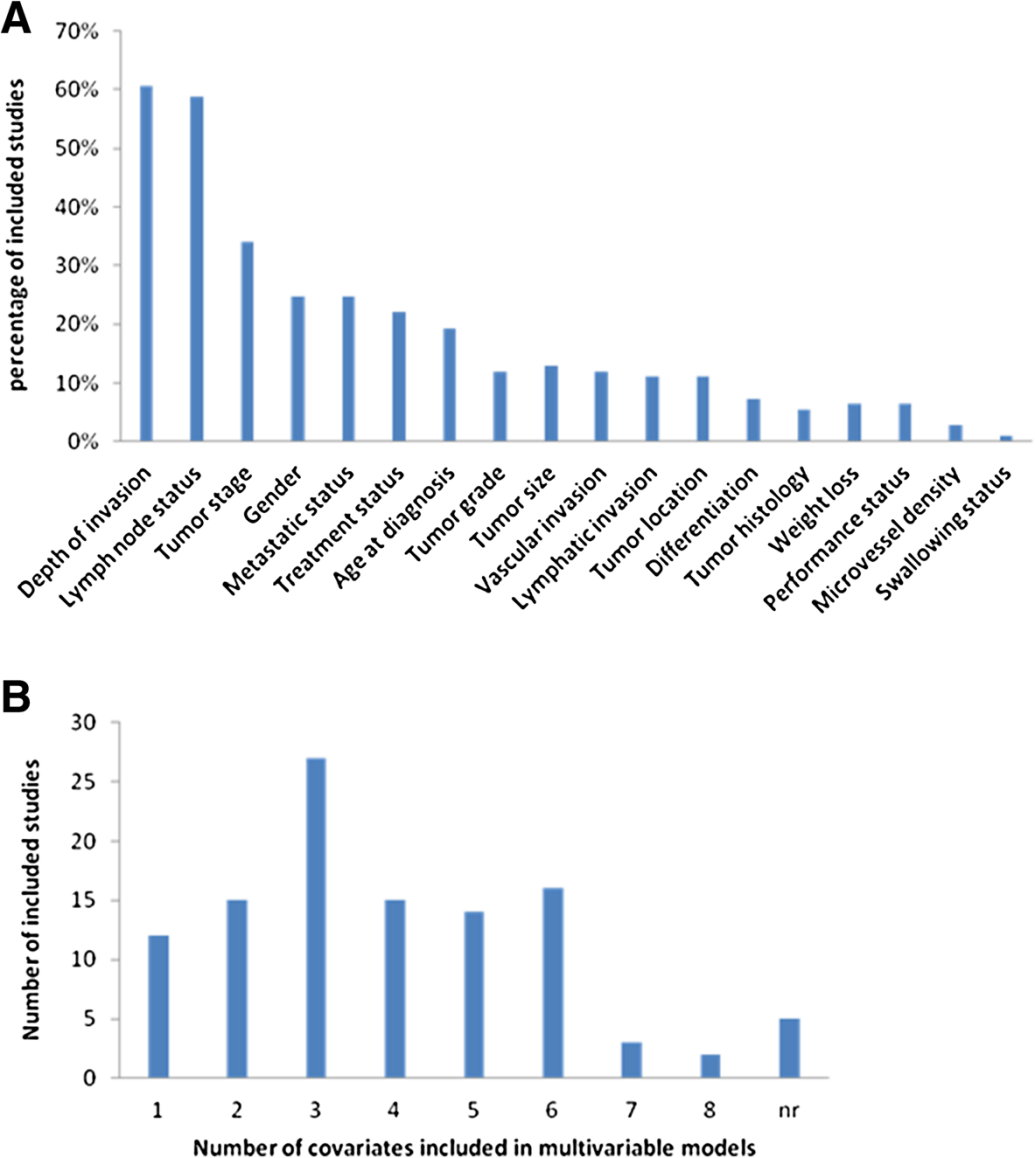
**Characteristics of included studies in the systematic review. A**. Frequencies with which adjustments were made for various clinicopathologic parameters. **B**. Distributions of the total number of clinicopathologic covariates that were adjusted for across the 109 eligible studies. NOTE: nr, not report.

All reported the prognostic value of biomarkers in patients with EC by presenting multivariable survival estimates for differential levels of candidate biomarker expression. Effective sample size ranged from 29 [18] to 708 patients [19] (median, 87 patients), with 13 studies including 50 or fewer patients, 54 studies including 51-100 individuals, 26 studies including 101-150 individuals, 12 studies including 151-300 individuals and 4 studies including more than 300 individuals. Seventeen clinicopathologic factors were incorporated in one or more of the eligible studies’ multivariate analysis. The most commonly included prognostic factor was depth of invasion involvement with lymph node status being included in 67 (61%) studies and 65 (59%) studies. Other common adjustment parameters included tumor stage (37 of 109 studies), gender (27 of 109 studies) and metastatic status (27 of 109 studies) (Figure 2A). Fifty-seven studies considered three to five clinical parameters in their multivariable proportional hazards models, 26 studies considered less than three covariates, 21 studies included more than five covariates and another 5 studies did not report(Figure 2B).

These 109 studies presented data on 13 unique biomarkers. The majority of eligible studies (n = 87) restricted their analysis to a single included candidate marker, and the remaining 22 evaluated between two to five markers. Regarding angiogenesis, 6 studies of cyclooxygenase-2 (COX-2) [20-25] and 19 studies of vascular endothelial growth factor (VEGF) [26-44] were included. Of the eligible marker associated with apoptosis, 5 studies of survivin [45-49] were available for analysis. For cell-cycle regulators, 9 studies of p21 [27,50-57] and 7 studies of p27 [25,58-63] were included. Four markers associated with replicative potential were eligible for examination, and were comprised of 15 studies of cyclin D1 [27,51,54,58,59,62,64-72], 32 eligible studies of p53 [27,30,32,34,38,40,51,54],[68,73-95], 6 studies of human epidermal growth factor receptor-2 (HER-2) [19,96-100] and 5 studies of Ki67 [25,27,87,101,102]. Regarding the tissue invasion and metastasis markers evaluated, 10 studies of E-cadherin [27,65,102-109] were included. Three eligible serum markers were eligible for analysis, and were comprised of 8 studies of C reative protein (CRP) [110-117], 5 studies of SCC-Ag [18,118-121] and 5 studies of hemoglobin (Hb) [122-126]. The 13 biomarkers were evaluated for overall survival (OS) and sorted according to five to eight Hanahan-Weinberg functional capabilities modified to include serum markers (Additional file 2: Table S2). Additional file 2: Table S2 outlines the demographic, clinicopathological, methodological and outcome characteristics of these studies.

### Quality of study reports

**Figure 3:**
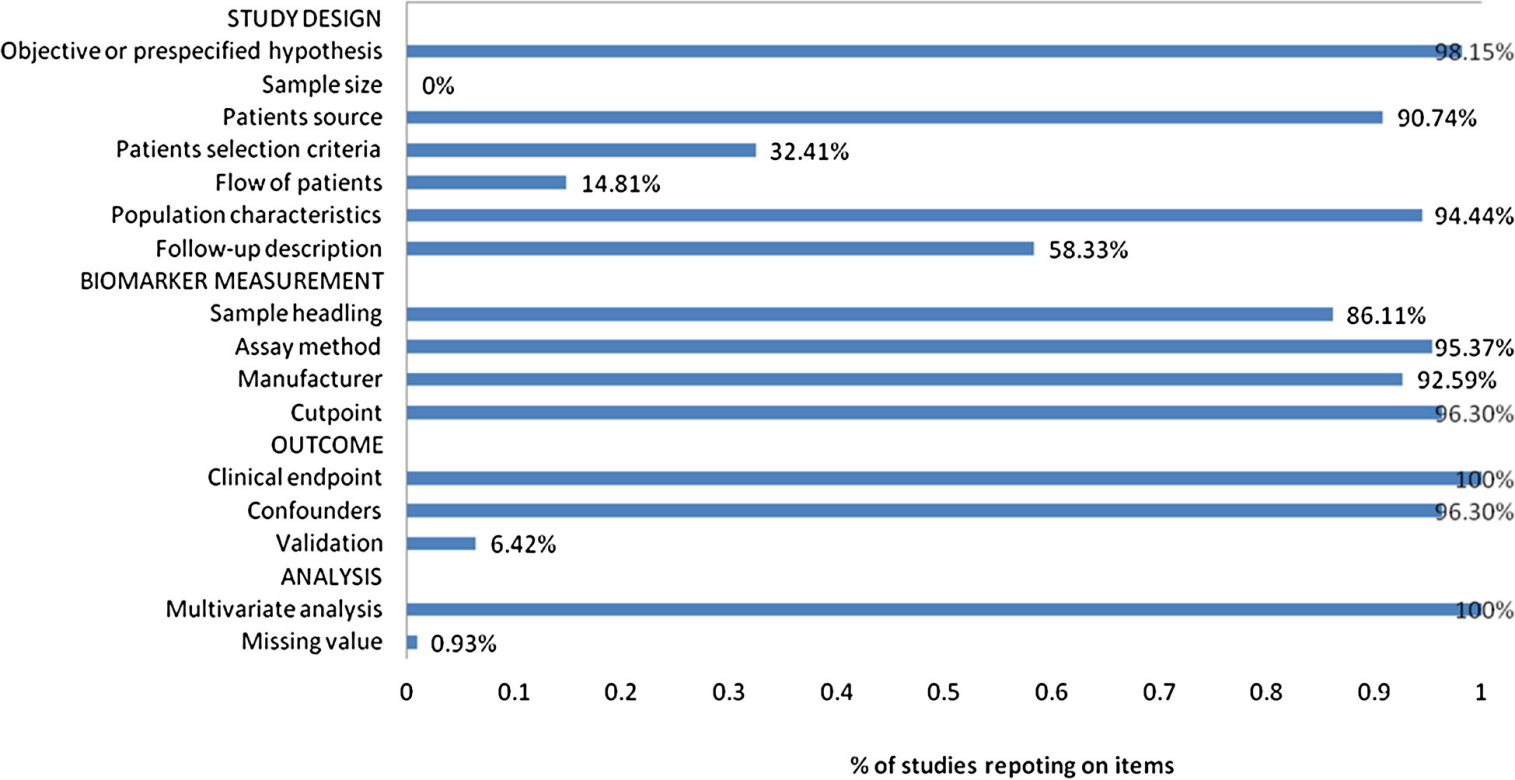
**Quality of individual study reports (n = 16 items, n = 109 studies), based on the REMARKER guidelines.** Definition items of each item are given in Additional file 1: Table S1.

The mean number of study quality items reported was 11 out of possible 16 and was not associated with sample size with correlation coefficient of 0.06, p = 0.52 (Figure 3). There was also no statistically significant difference between the quality items of 65 positive studies (where a biomarker was statistically a factor for poor prognosis) and 44 negative studies (where a biomarker was not statistically significant) (Mann-Whitney p = 0.27). All of the studies reported details of the clinical endpoint and multivariate analysis. More than 90% of studies reported details of the objective or prespecified hypothesis, patient source, population characteristics, assay method, manufacturer, cutpoint and confounders. Of note, 63 studies reported the follow-up period or the median follow-up time. One study referred to a missing value, but no study referred to a statistical sample size. To try to evaluate the impact of study quality on the final pooled estimate, a subgroup analysis was performed according to the different number of quality items: eleven or more eleven, which is the mean number of study quality items, and less than eleven. For majority markers, the results were consistent, the pooled HRs were not significantly altered, suggesting the study quality improbable as source of bias. For COX-2, only one study with quality items less than eleven, the HR was reported 2.34 (95% CI, 1.11-4.91). The pooled HR estimated for the other five studies was 1.36 (95% CI, 0.60-3.08), which is similar to the final pooled HR 1.54 (95% CI, 0.80-2.98). For HER-2, both subgroups included three studies eligible for meta-analysis, with combined HR 1.64 (95%CI,1.07-2.51) and 1.26 (95% CI, 0.70-2.26), respectively. Because of a small number of studies included, the results should be treated with caution.

### Meta-analysis results for biomarkers

For the 13 markers, a multivariate HR and 95% CI were available from five or more studies and were combined using both fixed effects general inverse variance and DerSimonian-Laird random effects modeling to obtain a single summary HR and 95% CI. (Table 1) Five of the six original Hanahan-Weinberg functional capabilities along with the additional group were represented by at least one marker statistically associated with OS. Two studies [35,91] were excluded which failed to present validated data in their meta-analysis.

**Table 1:** Summary of the multivariable hazard ratios and 95% CI for eligible biomarkers, organized according to the Hanahan-Weinberg functional capability

**Biomarker**	**Group**	**No. of studies**	**Patients**	**I-V HR(95% CI)**	**D + L HR(95% CI)**	**I**^**2**^**(%)**
**Angiogenesis**						
**COX-2**	Total(IHC)	6	469	2.00(1.47-2.71)	1.54(0.80-2.98)	75.0
	ESCC	4	234	1.23(0.79-1.93)	0.96(0.39-2.41)	73.5
	EADC	2	235	3.06(2.01-4.65)	3.06(2.01-4.65)	0.0
**VEGF**	Total	18	1476	1.80(1.51-2.14)	1.76(1.38-2.24)	43.5
	ESCC	16	1329	1.85(1.55-2.21)	1.84(1.45-2.33)	38.4
	EADC	1	38	0.37(0.10-1.40)	0.37(0.10-1.40)	-
	EC	1	109	1.80(0.70-4.65)	1.80(0.70-4.65)	-
	IHC	14	1109	1.59(1.28-1.98)	1.56(1.16-2.12)	43.6
	ELISA	2	179	2.55(1.72-3.79)	2.67(1.57-4.54)	36.8
	RTPCR	2	188	1.90(1.24-2.91)	1.90(1.24-2.91)	0.0
**Evading apoptosis**						
**survivin**	Total	5	357	1.60(1.23-2.07)	1.90(1.06-3.40)	74.6
	ESCC(IHC)	4	295	1.50(1.15-1.95)	1.57(0.91-2.69)	70.7
	EC(PCR)	1	62	6.60(1.97-22.12)	6.60(1.97-22.12)	-
**Insensitivity to antigrowth signal**						
**p21**	Total(IHC)	9	858	0.90(0.75-1.08)	1.27(0.75-2.16)	86.4
	ESCC	7	683	0.90(0.74-1.09)	1.28(0.70-2.33)	87.4
	EC	2	175	0.94(0.57-1.53)	1.31(0.22-7.98)	91.2
**p27(-)**	Total(IHC)	7	606	1.44(1.07-1.92)	1.68(0.90-3.12)	76.4
	ESCC	6	478	1.75(1.25-2.44)	1.97(1.00-3.88)	74.6
	EC	1	128	0.75(0.41-1.38)	0.75(0.41-1.38)	-
**Limitless replicative potential**						
**cyclin D1**	Total	15	1931	1.65(1.41-1.93)	1.73(1.34-2.23)	56.3
	ESCC	12	1295	1.82(1.50-2.20)	1.89(1.44-2.48)	46.2
	EC	3	636	1.37(1.05-1.80)	1.18(0.57-2.45)	77.4
	IHC	13	1735	1.60(1.36-1.88)	1.64(1.26-2.14)	57.9
	PCR	2	196	2.62(1.41-4.89)	2.79(1.27-6.14)	18.5
**HER-2**	Total	6	1162	1.06(0.88-1.28)	1.37(0.91-2.07)	67.6
	ESCC	1	66	0.92(0.35-2.41)	0.92(0.35-2.41)	-
	EADC	3	291	2.15(1.39-3.33)	2.15(1.39-3.33)	0.0
	EC	2	805	0.91(0.73-1.12)	0.93(0.70-1.23)	32.3
	IHC	4	951	0.96(0.78-1.17)	1.17(0.72-1.88)	66.3
	FISH	1	124	1.80(0.90-3.60)	1.80(0.90-3.60)	-
	RPPA	1	87	1.97(1.01-3.83)	1.97(1.01-3.83)	-
**Ki-67**	Total(IHC)	5	424	0.84(0.59-1.20)	0.76(0.41-1.42)	62.2
	ESCC	3	308	1.11(0.70-1.78)	1.11(0.70-1.78)	0.0
	EADC	1	59	0.26(0.11-0.60)	0.26(0.11-0.60)	-
	EC	1	57	1.03(0.49-2.17)	1.03(0.49-2.17)	-
**P53**	Total	31	2851	1.34(1.21-1.48)	1.33(1.14-1.56)	48.7
	ESCC	20	2063	1.26(1.11-1.42)	1.25(1.03-1.51)	48.7
	EADC	2	97	2.10(1.10-4.03)	2.10(1.10-4.03)	0.0
	EC	9	691	1.53(1.25-1.86)	1.44(1.06-1.94)	50.9
	IHC	22	2122	1.25(1.12-1.40)	1.20(1.02-1.40)	38.5
	PCR-SSCP	5	383	1.75(1.24-2.49)	1.82(0.93-3.56)	70.5
	ELISA	4	346	2.13(1.46-3.09)	2.13(1.46-3.09)	0.0
**Tissue invasion and metastasis**						
**E-cadherin(-)**	Total	10	1569	1.13(1.06-1.21)	1.30(1.07-1.58)	61.8
	ESCC	7	977	1.12(1.05-1.19)	1.24(0.99-1.56)	67.2
	EADC	1	59	3.30(0.99-10.99)	3.30(0.99-10.99)	-
	EC	2	533	1.41(1.05-1.89)	1.41(1.05-1.89)	0.0
	IHC	9	1472	1.28(1.11-1.49)	1.39(1.08-1.80)	60.4
	ELISA	1	97	1.10(1.02-1.18)	1.10(1.02-1.18)	-
**Serum markers**						
**SCC-Ag**	Total(ESCC/EIA)	5	700	1.28(0.97-1.69)	1.28(0.93-1.76)	16.4
	Total	8	1382	1.43(1.27-1.61)	2.65(1.64-4.27)	85.8
	ESCC	3	260	2.05(1.33-3.17)	2.77(1.12-6.86)	68.4
	EC	5	1122	1.39(1.23-1.57)	2.66(1.44-4.92)	90.1
	LPIA	1	262	3.30(2.17-5.01)	3.30(2.17-5.01)	-
	IHC	2	110	4.33(2.02-9.24)	4.33(2.02-9.24)	0.0
	ELISA	1	150	1.42(0.83-2.42)	1.42(0.83-2.42)	-
	CRP-kit	1	356	1.52(1.05-2.21)	1.52(1.05-2.21)	-
	LEHIA	1	291	1.18(1.03-1.36)	1.18(1.03-1.36)	-
	INA	1	123	12.12(3.45-42.57)	12.12(3.45-42.57)	-
	ITA	1	90	5.07(1.92-13.41)	5.07(1.92-13.41)	-
	Total	5	544	0.96(0.95-0.98)	0.91(0.83-1.00)	87.1
	ESCC	2	351	0.54(0.40-0.74)	0.54(0.40-0.74)	0.0
	EC	3	193	0.97(0.95-0.98)	0.96(0.89-1.03)	88.3

NOTE: HR, hazard ratio; CI, confidence interval. Combined HRs are calculated for the fixed effects general inverse variance (I-V) method and random effects model with DerSimonian and Laird (D + L) method. (-), reduced/negative expression show the higher risk in prognosis. IHC, immunohistochemistry; PCR, polymerase chain reaction; SSCP, single-strand conformation polymorphism; ELISA,enzyme-linked immunosorbent assay; LPIA, latex photometric immunoassay; LEHIA, latex-enhanced homogeneous immunoassay; INA, immunonephelometry; ITA, immunoturbidimetry; RIA, radioimmunoassay; EIA, enzyme immunoassay.

### COX-2, VEGF

Of the six studies that used immunohistochemistry (IHC) for OS data, the pooled HR was 1.54 (95% CI, 0.80-2.98), with significant evidence of heterogeneity between the contributing studies (I^2^ = 75%). Restricting analysis to the four studies assessing COX-2 expression in ESCC, the pooled HR was 0.96 (95% CI,0.39-2.41), again, with evidence of study heterogeneity (73.5%). These results should, however, be interpreted with caution because of the small number of contributing studies and the significant evidence for significant study heterogeneity. Two studies assessed COX-2 expression in EADC displayed a pooled HR of 3.06 (95% CI, 2.01-4.65), with no evidence of heterogeneity (I^2^ = 0.0).

**Figure 4:**
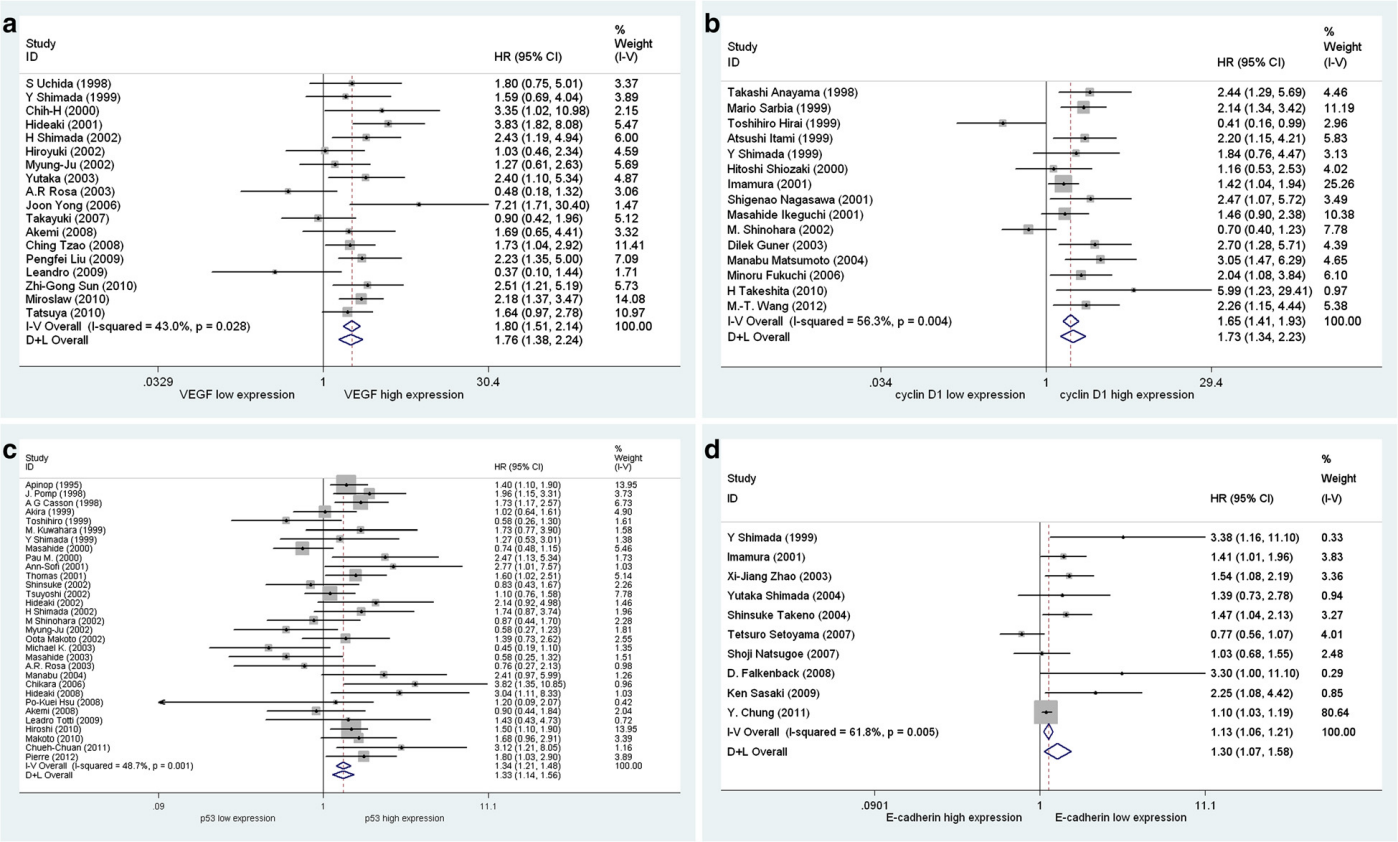
**Forest plots of the data for four biomarker-outcome comparisons for which eligible data were presented in ten or more studies.** Forest plots of HR for OS of **(A)** VEGF, **(B)** cyclin D1, **(C)** p53 and **(D)** E-cadherin. For each study, the hazard ratio (HR), 95% confidence interval (CI), and relative weight are show. Combined fixed effect HRs and tests for heterogeneity (I^2^) were based on the generic inverse variance (I-V) method. Combined random effect HRs were calculated according to the DerSimonian-Laird (D + L) method.

Of the eighteen VEGF expression studies eligible for pooling of OS data, the pooled HR was 1.80 (95% CI, 1.51-2.14) with no evidence of heterogeneity. The Forrest plot for this analysis is shown in Figure 4A. When restricting analysis to the sixteen studies examining VEGF expression in ESCC, the combined HR was 1.85 (95% CI, 1.55-2.21) with no evidence of heterogeneity. Of the other two studies, one presented data on EC and one on EADC, respectively. To assess the effect of four methods on evaluating VEGF expression, we pooled HRs from studies using IHC, enzyme-linked immunosorbent assay (ELISA) or reverse transcription polymerase chain reaction (RTPCR). ELISA-based studies demonstrated a larger pooled HR (HR 2.55, 95% CI, 1.72-3.79) compared to IHC-based studies (HR 1.59, 95% CI, 1.28-1.98) or RTPCR-based (HR 1.90, 95% CI, 1.24-2.91) studies.

### Survivin

For five studies evaluating survivin expression in esophageal cancers, the combined HR was 1.90 (95% CI, 1.06-3.40) and there was evidence for heterogeneity (I^2^ = 74.6%). The pooled HR estimated for survival in the four IHC-based studies involving ESCC was 1.57 (95% CI, 0.91-2.69), again, with significant evidence of heterogeneity (I^2^ = 70.7%). The remaining PCR-based study involved EC, and had an HR of 6.60 (95% CI, 1.97-22.12).

### p21, p27

Nine studies examined p21 and seven studies assessed p27 levels with the pooled HRs of 1.27 (95% CI, 0.75-2.16) and 1.68 (95% CI, 0.90-3.12), respectively, and there was evidence of heterogeneity within both groups. All studies used IHC to estimate the correlation between biomarker expression and survival. Interestingly, when grouped according to the histology of individual studies, the combined HR in ESCC for p21 (seven studies), remained unchanged at 1.28 (95% CI, 0.70-2.33). The pooled HR for p27 (six studies) in ESCC was 1.97 (95% CI, 1.00-3.88). In both analyses, there was significant evidence of study heterogeneity (I^2^ = 87.4% for p21 and I^2^ = 74.6% for p27, respectively).

### Cyclin D1, HER-2, Ki-67, p53

Fifteen studies assessed cyclin D1. The overall pooled HR was 1.73 (95% CI, 1.34-2.23) and there appeared to be some heterogeneity between the studies (I^2^ = 56.3%) Figure 4B illustrates the Forrest plot for the pooled data. In subgroup analysis, the pooled HR for ESCC was 1.82 (95% CI, 1.50-2.20) with no evidence of heterogeneity. Only three of the fifteen studies presented data evaluable for assessment of EC and the pooled HR was 1.18 (95% CI, 0.57-2.45). However, this result should be interpreted with caution because of the small number of contributing studies and evidence for significant study heterogeneity (I^2^ = 77.4%). To assess the effect of the method used to assess cyclin D1 expression, HRs, using either IHC or PCR, were pooled. This presented a large pooled HR for PCR-based studies (HR 2.62, 95% CI, 1.41-4.89) compared to that from the IHC-based studies (HR, 1.64, 95% CI, 1.26-2.14). The IHC-based group displayed significant heterogeneity, whereas the PCR-based group did not.

Six studies examined the HER-2 as a biomarker. When conducting subgroup analysis, we found three of six eligible studies assessed HER-2 expression in EADC and had a pooled HR of 2.15 (95% CI, 1.39-3.33) with no evidence of heterogeneity. Two studies assessed HER-2 expression in an EC setting with a pooled HR of 0.91 (95% CI, 0.73-1.12). Another study examined HER-2 in ESCC and reported an HR of 0.92 (95% CI, 0.35-2.41). Because of the small number of studies included, the results should be treated with caution.

Three of five studies eligible for assessing Ki-67 in ESCC demonstrated a pooled HR of 1.11 (95% CI, 0.70-1.78) with no significant of heterogeneity. None of the HR reported were statistically significant except in one study that assessed in EADC and reported an HR of 0.26 (95% CI, 0.11-0.60).

Thirty-one studies assessed p53. The pooled HR of 1.34 (95% CI, 1.21-1.48) revealed significant association with overall survival and the Forest plot for this analysis is shown in Figure 4C. Restricting analysis to the twenty studies assessing p53 expression in ESCC gave a pooled HR of 1.26 (95% CI, 1.11-1.42), with ELISA-based studies giving a larger pooled HR (2.13, 95% CI, 1.46-3.09) than IHC-based studies (HR, 1.25, 95% CI, 1.12-1.40).

### E-cadherin

Ten studies assessing the E-cadherin biomarker displayed a pooled HR of 1.13 (95% CI, 1.06-1.21) with evidence of heterogeneity (Forrest plot in Figure 4D). When restricting analysis to the seven studies examing E-cadherin in ESCC, however, the result remained unchanged, with an HR of 1.12 (95% CI, 1.05-1.19), again, with significant heterogeneity. For the two studies assessing E-cadherin expression in EC, the pooled HR was 1.41 (95% CI, 1.05-1.89), and there appeared to be no heterogeneity between the studies. An EADC study reported an HR of 3.30 (95% CI, 0.99-10.99). When grouped according to method, the combined HR of IHC-based studies was 1.28 (95% CI, 1.11-1.49) with significant heterogeneity. The only ELISA-based study reported an HR of 1.10 (95% CI, 1.02-1.18).

### SCC-Ag, CRP, Hb

Five eligible studies assessed SCC-Ag expression by enzyme immunoassay (EIA) in ESCC, and the pooled HR for OS was 1.28 (95% CI, 0.97-1.69) with no evidence of heterogeneity. Eight studies examined CRP expression with pooled HR 2.65 (95% CI, 1.64-4.27) and there was a significant heterogeneity. When conducted subgroup analysis, both EC and ESCC group still showed evidence of heterogeneity. When grouped according to different method used to assess CRP expression, two IHC-based studies had pooled HR 4.33 (95% CI, 2.02-9.24). Other studies used different method reported HR revealing a significant association with poor survival but one ELISA-based study (HR, 1.42, 95% CI, 0.83-2.42).

Five studies assessed Hb. The pooled HR was 0.91 (95% CI, 0.83-1.00) with significant heterogeneity. When restricting analysis to the three studies assessing Hb levels in EC, the pooled HR was 0.96 (95% CI, 0.89-1.03), again, with evidence of heterogeneity. Two studies assessing Hb levels in ESCC gave a pooled HR of 0.54 (95% CI, 0.40-0.74) with no evidence of heterogeneity. Only one included study reported no significant association with outcome. However, data were not sufficient to determine the prognostic value of Hb expression in either ESCC or EADC.

### Sensitivity analyses

We performed sensitivity analyses, in which one study was removed at a time, to evaluate the result stability. For COX-2, VEGF, cyclin D1, p53, E-cadherin and SCC-Ag, the results indicated that fixed-effects estimates and/or random effects estimate before and after the deletion of each study were similar at large, suggesting high stability of the meta-analysis results. For survivin and CRP, although the results are consistent with the overall pooled estimates, the influencing single study conducted by S. Mega et al. and Ines Gockel et al. respectively. For other markers, the sensitivity analysis did not indicate high stability of the results due to one or two studies. The results of sensitivity analyses are the supplement of the subgroup analysis results.

### Publication bias

Begg’s test and Egger’s test were used to examine publication bias. There was evidence for significant publication bias with p21 (Egger’s test p = 0.040), HER-2 (Egger’s test p = 0.042) and CRP (Egger’s test p = 0.005).

## Discussion

In response to the need for independent prognostic biomarkers for EC that are readily evaluated on routinely acquired clinical specimens, we performed a systematic review and meta-analysis of the published EC literature to identify the molecular markers for which the data support validation as prognostic biomarkers of EC outcome. Using stringent inclusion and exclusion criteria, examining patient selection, and evaluating both laboratory and statistical methodology [9], we identified 109 high-quality studies describing multivariate survival analysis for 13 unique biomarkers. Individual biomarker assay data were organized according to OS, and according to the Hanahan and Wernberg functional groupings that reflect the acquired capabilities of cancer as defined [12].

Quality assessment tools have been developed for prognostic studies to help identify study bias and causes of heterogeneity when performing meta-analysis. We chose to use and operationalize the REMARK reporting guidelines, which provide a useful start for assessing tumor prognostic biomarkers. We find that the studies reported an average of 11 of 16 quality items. Comparison of the quality items of positive and negative studies show no statistically significant difference, allowing meaningful data aggregation. Although this is a relatively new tool, it has been used in other system reviews [127,128].

We demonstrate that COX-2 and VEGF, regulators of angiogenesis, influence overall mortality, indicating the importance of this functional grouping in EC progression. Elevated COX-2 levels may be associated with worse outcome in EADC. Results in the present study demonstrate that, because variability between studies as to be the relative prognostic impact of COX-2 expression in ESSC, the observed survival trend in EADC is concordant with that reported for other malignancies [129]. Because VEGF is a heavily studied marker, the combined HR suggests that VEGF over-expression influences OS. This result is concordant with existing reports, which implicate a similar prognostic value for VEGF expression in other malignancies [130,131], and lends further weight to the assertion that angiogenesis is a key determinant in driving EC progression.

Survivin, a strong negative regulator of apoptosis, inhibits or prevents the activation of caspases and promotes cellular survival under otherwise apoptotic conditions [132]. Elevated levels of survivin are significantly associated with poor outcome in multiple studies, as judged by a random effect model.

Cyclin-dependent kinase inhibitors (CKIs) block G1/S phase transition, and decreased expression is thought to result in deregulated growth, promoting tumor progression [133]. Reduced expression of p27 has been shown to be a negative prognostic factor in many malignancies [134]. In the six studies included here, the combined HR demonstrates a significant association between decreased p27 and poor prognosis in ESCC. In contrast, we did not find expression of p21, a p53-inducible universal CKI, to be associated with OS similar to findings by Jaudah and colleagues, who find no correlation between expression of p21 and overall survival in colorectal cancer [135].

Among the four markers associated with limitless replicative potential, cylin D1 and p53 are the most consistently associated with OS. Cyclin D1 is an important cell cycle regulator, being one of the cyclin-dependent kinases, and is controlled from chromosomal region 11q13, which is known to undergo amplification in several cancers, including head and neck cancer. Results again showed a significantly increased likelihood of poor prognosis for ESCC patients when positive for this biomarker. For p53, the finding that positive expression represents a favorable prognostic feature is consistent with its tumor suppressor function. For Ki-67, a proliferation-associated nuclear protein, only one of five included studies identifies a significant association, failing to support a prognostic role in EC patients, whereas HER-2, a member of epidermal growth factor receptor family, shows association with OS in EADC.

In terms of functional capabilities, markers, involved in facilitating tissue invasion and metastasis, include E-cadherin, whose disappearance is a hallmark of epithelial-mesenchymal transition [136]. E-cadherin is down-regulated in several epithelial malignancies [137]. Ten eligible studies, analyzed here by random effects modeling, further support the prognostic role of E-cadherin loss in OS.

SCC-Ag is a tumor-associated antigen and was originally isolated from a squamous cell carcinoma (SCC). Five studies relating to prognosis in ESSC do not reveal elevated SCC-Ag to be associated with poor survival. The current findings are in agreement with reports for SCC-Ag in patients with cancers of the cervix and lung [138,139]. CRP has been shown to be of prognostic value in many malignancies [140]. We further show that high CRP expression is significantly correlated with poor survival in EC. We report on five studies concerning serum Hb levels, four of which find that a reduced Hb level has a significant relationship with decreased survival. Overall, the evidence suggests that serum Hb measurement is a significant prognostic marker, but the strength of correlation is poor.

The strength of our study lies in the broad, unbiased survey of the available esophageal cancer literature and application of standard systematic review and meta-analysis methods to objectively identify manuscripts with robust data for summarization. However, there are several limitations inherent to our study. By not evaluating redundant study data, we attempted to avoid repeated inclusion of manuscripts from different publications, and focus on studies assessing prognostic markers with standard oncological endpoints of overall survival, while excluding studies with evaluation of recurrence. This study is also limited from the perspective that, for 13 of the eligible markers, summary data across outcome was derived from association data presented in more than four studies, excluding markers represented in included studies fewer than five.

For the included studies, the across-study heterogeneity in the execution of test methods as well as categorization and statistical adjustments for the clinicopathologic factors included in our multivariate analysis may contribute to measurement error of the biomarker to outcome associations. Although most of the authors corrected for established prognostic variables, variations included in adjustment method contribute to inaccuracy related to risk estimation. Variability in assessment of marker expression and cuto-ff point selection across studies may be considered as a potential source of bias. For majority of markers, selection of cut-point to categories marker expression was arbitrary and varied among studies even for the same marker using the same kind of test method. For markers included more than ten studies (VEGF, cyclin D1, p53) for overall survival estimate, stratified analyses was performed according to cut-point value. For VEGF, the choice of the cutoff value for VEGF positivity in IHC (14 studies) varied from 10 to 80% among studies. Seven studies used 10% with combined HR 2.03 (95% CI, 1.51-2.73), this finding is consistent with the pooled HR 1.80 (95% CI, 1.51-2.14). In the other groups, the number of studies eligible for estimate is less than five and heterogeneity are significant, so the results should be considered with caution. For cyclin D1 and p53, the situation is similar. Only 10% group with five or more studies eligible for meta-analysis. Adoption of consensus cutpoints across the esophageal cancer community could facilitate replication of results. Additional studies with consistent methodology are needed to define the precise prognostic value of biomarkers.

Publication bias remains a problem in assessing the validity of research studies. Although the power to detect publication bias is reduced when fewer studies are included, when using the Egger’s test on our meta-analysis, 10 of 13 biomarkers do not show evidence that publication bias significantly influenced the results. However, analysis of 3 biomarkers (p21, HER-2 and CRP) did display significant publication bias. This may possibly be due to missing data because of unpublished studies. Our review takes into account only published studies. We did not search unpublished studies and abstracts because the methodology we used requires data that are usually only available in full publication studies. Missing information may reflect a negative or more conservative correlation between markers and survival, which could lower the significance of markers expression as a predictor of mortality [141]. Thus, the results for p21, HER-2 and CRP should be treated with considerable caution.

## Conclusions

Research in EC has identified a multitude of molecular markers with a significant role in predicting outcome. In this review, despite the inherent limitations of meta-analysis on prognostic literature, we identify several biomarkers of particular interest that appear to carry prognostic significance. Of 13 biomarkers analyzed, we find VEGF, cyclin D1, Ki-67, and SCC-Ag appeared to hold potential as predictors of outcome in ESCC; COX-2 and HER-2 in EADC; and p21, p53, CRP and Hb in EC. Several biomarkers did not have sufficient data for determination of prognostic value in esophageal cancers. There is a need for biomarker expression and validation of these potential markers in large cohorts of patients. Additional studies with consistent methodology are needed to define the precise prognostic value of biomarkers.

## Competing interests

The authors declare that they have no competing interest.

## Authors’ contributions

Conception and design: KL and MC; Acquisition of data: all authors; Analysis and interpretation of data: all authors; Manuscript drafting: MC and KL; Manuscript revising: all authors; final approval of this version: all authors. All Authors read and approved the final manuscript.

## Pre-publication history

The pre-publication history for this paper can be accessed here:

http://www.biomedcentral.com/1471-2407/13/539/prepub

## Supplementary Material

Additional file 1: Table S1 Definitions of 16 items of study reporting quality. Click here for file

Additional file 2: Table S2 The characteristics of the eligible studies for this systematic review. Click here for file
