# Erratum to: - Systematic review and meta-analysis of tumor biomarkers in predicting prognosis in esophageal cancer

**DOI:** 10.1186/1471-2407-14-892

**Published:** 2014-12-03

**Authors:** Meilan Chen, Jizheng Huang, Zhenli Zhu, Jun Zhang, Ke Li

**Affiliations:** Department of Preventive Medicine, Shantou University Medical College, No.22 Xinling Road, Shantou, Guangdong 515041 China

## Correction

After publication of this article ( [1]) we noted errors in the references. The term ‘Fau’ with varying initials has been included amongst the names of the authors in about a half of the citations, while the fist author of these articles has been omitted. A corrected reference list can be found below:

1. Jemal A, Bray F, Center MM, Ferlay J, Ward E, Forman D: **Global cancer statistics.** *CA Canc J Clin* 2011, **61:**69–90.
2. Kollarova H, Machova L, Horakova D, Janoutova G, Janout V: **Epidemiology of esophageal cancer–an overview article.** *Biomed Papers Med Faculty University Palacky Olomouc Czechoslovakia* 2007, **151:**17–20.
3. Simard EP, Ward EM, Siegel R, Jemal A: **Cancers with increasing incidence trends in the United States: 1999 through 2008. LID -**10.3322/caac.20141**[doi].** *CA Canc J Clin* 2012, **4:**20141.
4. Vial M, Grande L, Pera M: **Epidemiology of adenocarcinoma of the esophagus, gastric cardia, and upper gastric third.** *Recent Results Canc Res* 2010, **182:**1–17.
5. Gavin AT, Francisci S, Foschi R, Donnelly DW, Lemmens V, Brenner H, Anderson LA: **Oesophageal cancer survival in Europe: A EUROCARE-4 study.** *Canc Epidemiol* 2012, **36:**505–512.
6. Cen P, Banki F, Cheng L, Khalil K, Du XL, Fallon M, Amato RJ, Kaiser LR: **Changes in age, stage distribution, and survival of patients with esophageal adenocarcinoma over three decades in the United States.** *Ann Surg Oncol* 2012, **19:**1685–1691.
7. Merkow RP, Bilimoria KY, McCarter MD, Chow WB, Gordon HS, Stewart AK, Ko CY, Bentrem DJ: **Effect of histologic subtype on treatment and outcomes for esophageal cancer in the United States.** *Cancer* 2012, **118:**3268–3276.
8. Blom RL, Lagarde SM, Klinkenbijl JHG, Busch OR,van Berge Henegouwen MI: **A high body mass index in esophageal cancer patients does not influence postoperative outcome or long-term survival.** *Ann Surg Oncol* 2012, **19:**766–771. Epub 2011 Oct 2017.
9. McShane LM, Altman DG, Sauerbrei W, Taube SE, Gion M, Clark GM: **REporting recommendations for tumour MARKer prognostic studies (REMARK).** *Br J Cancer* 2005, **93:**387–391.
10. Altman DG: **Systematic reviews of evaluations of prognostic variables.** *BMJ* 2001, **323:**224–228.
11. Steels E, Paesmans M, Berghmans T, Branle F, Lemaitre F, Mascaux C, Meert AP, Vallot F, Lafitte JJ, Sculier JP: **Role of p53 as a prognostic factor for survival in lung cancer: a systematic review of the literature with a meta-analysis.** *Eur Respir J* 2001, **18:**705–719.
12. Hanahan D, Weinberg RA: **Hallmarks of cancer: the next generation.** *Cell* 2011, **144:**646–674.
13. DerSimonian R, Laird N: **Meta-analysis in clinical trials.** *Contr Clin Trials* 1986, **7:**177–188.
14. Higgins JP, Thompson SG, Deeks JJ, Altman DG: **Measuring inconsistency in meta-analyses.** *BMJ* 2003, **327:**557–560.
15. Begg CB, Mazumdar M: **Operating characteristics of a rank correlation test for publication bias.** *Biometrics* 1994, **50:**1088–1101.
16. Song F, Gilbody S: **Bias in meta-analysis detected by a simple, graphical test. Increase in studies of publication bias coincided with increasing use of meta-analysis.** *BMJ* 1998, **316:**471.
17. Egger M, Davey Smith G, Schneider M, Minder C: **Bias in meta-analysis detected by a simple, graphical test.** *BMJ* 1997, **315:**629–634.
18. Nabeya Y, Shimada H, Okazumi S, Matsubara H, Gunji Y, Suzuki T, Ochiai T: **Serum cross-linked carboxyterminal telopeptide of type I collagen (ICTP) as a prognostic tumor marker in patients with esophageal squamous cell carcinoma.** *Cancer* 2002, **94:**940–949.
19. Yoon HH, Shi Q , Sukov WR, Wiktor AE, Khan M, Sattler CA , Grothey A, Wu T-T, Diasio RB, Jenkins RB, Sinicrope FA: **Association of HER2/ErbB2 expression and gene amplification with pathologic features and prognosis in esophageal adenocarcinomas.** *Clin Cancer Res* 2012, **18:**546–554.
20. Buskens CJ, Van Rees BP, Sivula A, Reitsma JB, Haglund C, Bosma PJ, Offerhaus GJ, Van Lanschot JJ, Ristimaki A: **Prognostic significance of elevated cyclooxygenase 2 expression in patients with adenocarcinoma of the esophagus.** *Gastroenterology* 2002, **122:**1800–1807.
21. Nozoe T, Ezaki T, Kabashima A, Baba H, Maehara Y: **Significance of immunohistochemical expression of cyclooxygenase-2 in squamous cell carcinoma of the esophagus.** *Am J Surg* 2005, **189:**110–115.
22. Sivula A, Buskens CJ, van Rees BP, Haglund C, Offerhaus GJ, van Lanschot JJ, Ristimaki A: **Prognostic role of cyclooxygenase-2 in neoadjuvant-treated patients with squamous cell carcinoma of the esophagus.** *Int J Canc J Int Canc* 2005, **116:**903–908.
23. Bhandari P, Bateman AC, Mehta RL, Stacey BS, Johnson P, Cree IA, Di Nicolantonio F, Patel P: **Prognostic significance of cyclooxygenase-2 (COX-2) expression in patients with surgically resectable adenocarcinoma of the oesophagus.** *BMC Canc* 2006, **6:**134.
24. Yoon MS, Nam T-K, Lee J-S, Cho S-H, Song J-Y, Ahn S-J, Chung I-J, Jeong J-U, Chung W-K, Nah B-S: **VEGF as a predictor for response to definitive chemoradiotherapy and COX-2 as a prognosticator for survival in esophageal squamous cell carcinoma.** *J Korean Med Sci* 2011, **26:**513–520. Epub 2011 Mar 2028.
25. Huang JX, Chen WC, Lin M, Zhang YL, Li FY, Song ZX, Xiao W, Chen P, Qian RY, Salminen E, Yu H: **Clinicopathological significance of cyclooxygenase-2 and cell cycle-regulatory proteins expression in patients with esophageal squamous cell carcinoma.** *Dis Esophagus Off J Int Soc Dis Esophagus / ISDE* 2012, **25:**121–129.
26. Uchida S, Shimada Y, Watanabe G, Tanaka H, Shibagaki I, Miyahara T, Ishigami S, Imamura M: **In oesophageal squamous cell carcinoma vascular endothelial growth factor is associated with p53 mutation, advanced stage and poor prognosis.** *Br J Cancer* 1998, **77:**1704–1709.
27. Shimada Y, Imamura M, Watanabe G, Uchida S, Harada H, Makino T, Kano M: **Prognostic factors of oesophageal squamous cell carcinoma from the perspective of molecular biology.** *Br J Cancer* 1999, **80:**1281–1288.
28. Shih CH, Ozawa S, Ando N, Ueda M, Kitajima M: **Vascular endothelial growth factor expression predicts outcome and lymph node metastasis in squamous cell carcinoma of the esophagus.** *Clin Canc Res Offic J Am Assoc Canc Res* 2000, **6:**1161–1168.
29. Shimada H, Takeda A, Nabeya Y, Okazumi SI, Matsubara H, Funami Y, Hayashi H, Gunji Y, Kobayashi S, Suzuki T, Ochiai T: **Clinical significance of serum vascular endothelial growth factor in esophageal squamous cell carcinoma.** *Cancer* 2001, **92:**663–669.
30. Ahn MJ, Jang SJ, Park YW, Choi JH, Oh HS, Lee CB, Paik HK, Park CK: **Clinical prognostic values of vascular endothelial growth factor, microvessel density, and p53 expression in esophageal carcinomas.** *J Kor Med Sci* 2002, **17:**201–207.
31. Kato H, Yoshikawa M, Miyazaki T, Nakajima M, Fukai Y, Masuda N, Fukuchi M, Manda R, Tsukada K, Kuwano H: **Expression of vascular endothelial growth factor (VEGF) and its receptors (Flt-1 and Flk-1) in esophageal squamous cell carcinoma.** *Anticancer Res* 2002, **22:**3977–3984.
32. Shimada H, Hoshino T, Okazumi S, Matsubara H, Funami Y, Nabeya Y, Hayashi H, Takeda A, Shiratori T, Uno T, *et al.*: **Expression of angiogenic factors predicts response to chemoradiotherapy and prognosis of oesophageal squamous cell carcinoma.** *Br J Canc* 2002, **86:**552–557.
33. Ogata Y, Fujita H, Yamana H, Sueyoshi S, Shirouzu K: **Expression of vascular endothelial growth factor as a prognostic factor in node-positive squamous cell carcinoma in the thoracic esophagus: long-term follow-up study.** *World J Surg* 2003, **27:**584–589. Epub 2003 Apr 2028.
34. Rosa AR, Schirmer CC, Gurski RR, Meurer L, Edelweiss MI, Kruel CD: **Prognostic value of p53 protein expression and vascular endothelial growth factor expression in resected squamous cell carcinoma of the esophagus.** *Dis Esophagus Offic J Int Soc Dis Esophagus / ISDE* 2003, **16:**112–118.
35. Dreilich M, Wagenius G, Bergstrom S, Brattstrom D, Larsson A, Hesselius P, Bergqvist M: **The role of cystatin C and the angiogenic cytokines VEGF and bFGF in patients with esophageal carcinoma.** *Med Oncol* 2005, **22:**29–38.
36. Choi JY, Jang KT, Shim YM, Kim K, Ahn G, Lee KH, Choi Y, Choe YS, Kim BT: **Prognostic significance of vascular endothelial growth factor expression and microvessel density in esophageal squamous cell carcinoma: comparison with positron emission tomography.** *Ann Surg Oncol* 2006, **13:**1054–1062.
37. Kii T, Takiuchi H, Kawabe S, Gotoh M, Ohta S, Tanaka T, Kuwakado S, Nishitani H, Katsu K: **Evaluation of prognostic factors of esophageal squamous cell carcinoma (stage II-III) after concurrent chemoradiotherapy using biopsy specimens.** *Jpn J Clin Oncol* 2007, **37:**583–589.
38. Inoue A, Moriya H, Katada N, Tanabe S, Kobayashi N, Watanabe M, Okayasu I, Ohbu M: **Intratumoral lymphangiogenesis of esophageal squamous cell carcinoma and relationship with regulatory factors and prognosis.** *Pathol Int* 2008, **58:**611–619.
39. Tzao C, Lee S-C, Tung H-J, Hsu H-S , Hsu W-H, Sun G-H, Yu C-P, Jin J-S, Cheng YL: **Expression of hypoxia-inducible factor (HIF)-1alpha and vascular endothelial growth factor (VEGF)-D as outcome predictors in resected esophageal squamous cell carcinoma.** *Dis Markers* 2008, **25:**141–148.
40. Cavazzola L, Rosa ARP, Schirmer CC, Gurski RR, Telles JPB, Mielke F, Meurer L, Edelweiss MIA, Kruel CD: **Immunohistochemical evaluation for P53 and VEGF (Vascular Endothelial Growth Factor) is not prognostic for long term survival in end stage esophageal adenocarcinoma.** *Rev Col Bras Cir* 2009, **36:**24–34.
41. Liu P, Chen W, Zhu H, Liu B, Song S, Shen W, Wang F, Tucker S, Zhong B, Wang D: **Expression of VEGF-C correlates with a poor prognosis based on analysis of prognostic factors in 73 patients with esophageal squamous cell carcinomas.** *Jpn J Clin Oncol* 2009, **39:**644–650.
42. Kozlowski M, Kowalczuk O, Milewski R, Chyczewski L, Niklinski J, Laudanski J: **Serum vascular endothelial growth factors C and D in patients with oesophageal cancer.** *Eur J Cardio-thoracic Surg Offic J Eur Assoc Cardio-thoracic Surg* 2010, **38:**260–267.
43. Tanaka T, Ishiguro H, Kuwabara Y, Kimura M, Mitsui A, Katada T, Shiozaki M, Naganawa Y, Fujii Y, Takeyama H: **Vascular endothelial growth factor C (VEGF-C) in esophageal cancer correlates with lymph node metastasis and poor patient prognosis.** *J Exp Clin Canc Res: CR* 2010, **29:**83.
44. Sun ZG, Wang Z, Liu XY, Liu FY: **Mucin 1 and vascular endothelial growth factor C expression correlates with lymph node metastatic recurrence in patients with N0 esophageal cancer after Ivor-Lewis esophagectomy.** *World J Surg* 2011, **35:**70–77.
45. Mega S, Miyamoto M, Li L, Kadoya M, Takahashi R, Hase R, Kaneko H, Shichinohe T, Kawarada Y, Itoh T, Morikawa T, *et al.*: **Immunohistochemical analysis of nuclear survivin expression in esophageal squamous cell carcinoma.** *Dis Esophagus: Offic J Int Soc Dis Esophagus / ISDE* 2006, **19:**355–359.
46. Rosato A, Pivetta M, Parenti A, Iaderosa GA, Zoso A, Milan G, Mandruzzato S, Del Bianco P, Ruol A, Zaninotto G, Zanovello P: **Survivin in esophageal cancer: An accurate prognostic marker for squamous cell carcinoma but not adenocarcinoma.** *Int J Cancer* 2006, **119:**1717–1722.
47. Hsu KF, Lin CK, Yu CP, Tzao C, Lee SC, Lee YY, Tsai WC, Jin JS: **Cortactin, fascin, and survivin expression associated with clinicopathological parameters in esophageal squamous cell carcinoma.** *Dis Esophagus: Offic J Int Soc Dis Esophagus / ISDE* 2009, **22:**402–408.
48. Hoffmann AC, Vallbohmer D, Grimminger P, Metzger R, Prenzel KL, Hoelscher AH, Brabender J: **Preoperative survivin mRNA detection in peripheral blood is an independent predictor of outcome in esophageal carcinoma.** *Pharmacogenomics* 2010, **11:**341–347.
49. Takeno S, Yamashita S, Takahashi Y, Ono K, Kamei M, Moroga T, Kawahara K: **Survivin expression in oesophageal squamous cell carcinoma: its prognostic impact and splice variant expression.** *Eur J Cardio-thoracic Surg: Offic J Eur Assoc Cardio-thoracic Surg* 2010, **37:**440–445.
50. Sarbia M, Stahl M, zur Hausen A, Zimmermann K, Wang L, Fink U, Heep H, Dutkowski P, Willers R, Muller W, *et al.*: **Expression of p21WAF1 predicts outcome of esophageal cancer patients treated by surgery alone or by combined therapy modalities.** *Clin Canc Res Offic J Am Assoc Canc Res* 1998, **4:**2615–2623.
51. Hirai T, Kuwahara M, Yoshida K, Osaki A, Toge T: **The prognostic significance of p53, p21 (Waf1/Cip1), and cyclin D1 protein expression in esophageal cancer patients.** *Anticancer Res* 1999, **19:**4587–4591.
52. Natsugoe S, Nakashima S, Matsumoto M, Xiangming C, Okumura H, Kijima F, Ishigami S, Takebayashi Y, Baba M, Takao S, Aikou T: **Expression of p21WAF1/Cip1 in the p53-dependent pathway is related to prognosis in patients with advanced esophageal carcinoma.** *Clin Cancer Res* 1999, **5:**2445–2449.
53. Nita ME, Nagawa H, Tominaga O, Tsuno N, Hatano K, Kitayama J, Tsuruo T, Domene CE, Muto T: **p21Waf1/Cip1 expression is a prognostic marker in curatively resected esophageal squamous cell carcinoma, but not p27Kip1, p53, or Rb.** *Ann Surg Oncol* 1999, **6:**481–488.
54. Matsumoto M, Furihata M, Kurabayashi A, Sasaguri S, Araki K, Hayashi H, Ohtsuki Y: **Prognostic significance of serine 392 phosphorylation in overexpressed p53 protein in human esophageal squamous cell carcinoma.** *Oncology* 2004, **67:**143–150.
55. Nakamura T, Hayashi K, Ota M, Ide H, Takasaki K, Mitsuhashi M: **Expression of p21(Waf1/Cip1) predicts response and survival of esophageal cancer patients treated by chemoradiotherapy.** *Dis Esophagus* 2004, **17:**315–321.
56. Goan Y-G, Hsu H-K, Chang H-C, Chou Y-P, Chiang K-H, Cheng JT: **Deregulated p21(WAF1) overexpression impacts survival of surgically resected esophageal squamous cell carcinoma patients.** *Ann Thorac Surg* 2005, **80:**1007–1016.
57. Taghavi N, Biramijamal F, Sotoudeh M, Moaven O, Khademi H, Abbaszadegan MR, Malekzadeh R: **Association of p53/p21 expression with cigarette smoking and prognosis in esophageal squamous cell carcinoma patients.** *World J Gastroenterol: WJG* 2010, **16:**4958–4967.
58. Anayama T, Furihata M, Ishikawa T, Ohtsuki Y, Ogoshi S: **Positive correlation between p27Kip1 expression and progression of human esophageal squamous cell carcinoma.** *Int J Cancer* 1998, **79:**439–443.
59. Itami A, Shimada Y, Watanabe G, Imamura M: **Prognostic value of p27(Kip1) and CyclinD1 expression in esophageal cancer.** *Oncology* 1999, **57:**311–317.
60. Yasunaya M, Tabira Y, Kondo K, Okuma T, Kitamura N: **The prognostic significance of cell cycle markers in esophageal cancer after neoadjuvant chemotherapy.** *Dis Esophagus: Offic J Int Soc Dis Esophagus / ISDE* 1999, **12:**120–127.
61. Shamma A, Doki Y, Tsujinaka T, Shiozaki H, Inoue M, Yano M, Kawanishi K, Monden M: **Loss of p27(KIP1) expression predicts poor prognosis in patients with esophageal squamous cell carcinoma.** *Oncology* 2000, **58:**152–158.
62. Shiozaki H, Doki Y, Kawanishi K, Shamma A, Yano M, Inoue M, Monden M: **Clinical application of malignancy potential grading as a prognostic factor of human esophageal cancers.** *Surgery* 2000, **127:**552–561.
63. Nishioka K, Doki Y, Shiozaki H, Yamamoto H, Tamura S, Yasuda T, Fujiwara Y, Yano M, Miyata H, Kishi K, *et al.*: **Clinical significance of CDC25A and CDC25B expression in squamous cell carcinomas of the oesophagus.** *Br J Canc* 2001, **85:**412–421.
64. Sarbia M, Stahl M, Fink U, Heep H, Dutkowski P, Willers R, Seeber S, Gabbert HE: **Prognostic significance of cyclin D1 in esophageal squamous cell carcinoma patients treated with surgery alone or combined therapy modalities.** *Int J Canc* 1999, **84:**86–91.
65. Research Committee on Malignancy of Esophageal Cancer, Japanese Society for Esophageal Diseases: **Prognostic significance of CyclinD1 and E-Cadherin in patients with esophageal squamous cell carcinoma: multiinstitutional retrospective analysis.** *J Am Coll Surg* 2001, **192:**708–718.
66. Ikeguchi M, Sakatani T, Ueta T, Kaibara N: **Cyclin D1 expression and retinoblastoma gene protein (pRB) expression in esophageal squamous cell carcinoma.** *J Canc Res Clin Oncol* 2001, **127:**531–536.
67. Nagasawa S, Onda M, Sasajima K, Makino H, Yamashita K, Takubo K, Miyashita M: **Cyclin D1 overexpression as a prognostic factor in patients with esophageal carcinoma.** *J Surg Oncol* 2001, **78:**208–214.
68. Shinohara M, Aoki T, Sato S, Takagi Y, Osaka Y, Koyanagi Y, Hatooka S, Shinoda M: **Cell cycle-regulated factors in esophageal cancer.** *Dis Esophagus Offic J Int Soc Dis Esophagus / ISDE* 2002, **15:**149–154.
69. Guner D, Sturm I, Hemmati P, Hermann S, Hauptmann S, Wurm R, Budach V, Dorken B, Lorenz M, Daniel PT: **Multigene analysis of Rb pathway and apoptosis control in esophageal squamous cell carcinoma identifies patients with good prognosis.** *Int J Cancer* 2003, **103:**445–454.
70. Fukuchi M, Fukai Y, Kimura H, Sohda M, Miyazaki T, Nakajima M, Masuda N, Tsukada K, Kato H, Kuwano H: **Prolyl isomerase Pin1 expression predicts prognosis in patients with esophageal squamous cell carcinoma and correlates with cyclinD1 expression.** *Int J Oncol* 2006, **29:**329–334.
71. Takeshita H, Ichikawa D, Komatsu S, Tsujiura M, Kosuga T, Deguchi K, Konishi H, Morimura R, Shiozaki A, Fujiwara H, Okamoto K, *et al.*: **Prediction of CCND1 amplification using plasma DNA as a prognostic marker in oesophageal squamous cell carcinoma.** *Br J Canc* 2010, **102:**1378–1383. Epub 2010 Apr 1313.
72. Wang MT, Chen G, An SJ, Chen ZH, Huang ZM, Xiao P, Ben XS, Xie Z, Chen SL, Luo DL, Tang JM, *et al.*: **Prognostic significance of cyclinD1 amplification and the co-alteration of cyclinD1/pRb/ppRb in patients with esophageal squamous cell carcinoma.** *Dis Esophagus* 2012, **25:**664–670. doi:610.1111/j.1442-2050.2011.01291.x. Epub 02011 Dec 01299.
73. Chanvitan A, Nekarda H, Casson AG: **Prognostic value of DNA index, S-phase fraction and p53 protein accumulation after surgical resection of esophageal squamous-cell carcinomas in Thailand.** *Int J Canc J Int Canc* 1995, **63:**381–386.
74. Casson AG, Tammemagi M, Eskandarian S, Redston M, McLaughlin J, Ozcelik H: **p53 alterations in oesophageal cancer: association with clinicopathological features, risk factors, and survival.** *Mol Pathol* 1998, **51:**71–79.
75. Pomp J, Davelaar J, Blom J, van Krimpen C, Zwinderman A, Quint W, Immerzeel J: **Radiotherapy for oesophagus carcinoma: the impact of p53 on treatment outcome.** *Radiother Oncol J Eur Soc Ther Radiol Oncol* 1998, **46:**179–184.
76. Kanamoto A, Kato H, Tachimori Y, Watanabe H, Nakanishi Y, Kondo H, Yamaguchi H, Gotoda T, Muro K, Matsumura Y: **No prognostic significance of p53 expression in esophageal squamous cell carcinoma.** *J Surg Oncol* 1999, **72:**94–98.
77. Kuwahara M, Hirai T, Yoshida K, Yamashita Y, Hihara J, Inoue H, Toge T: **p53, p21(Waf1/Cip1) and cyclin D1 protein expression and prognosis in esophageal cancer.** *Dis Esophagus Offic J Int Soc Dis Esophagus / ISDE* 1999, **12:**116–119.
78. Ikeguchi M, Oka S, Gomyo Y, Tsujitani S, Maeta M, Kaibara N: **Combined analysis of p53 and retinoblastoma protein expressions in esophageal cancer.** *Ann Thorac Surg* 2000, **70:**913–917.
79. Schneider PM, Stoeltzing O, Roth JA, Hoelscher AH, Wegerer S, Mizumoto S, Becker K, Dittler HJ, Fink U, Siewert JR: **P53 mutational status improves estimation of prognosis in patients with curatively resected adenocarcinoma in Barrett's esophagus.** *Clin Canc Res Offic J Am Assoc Canc Res* 2000, **6:**3153–3158.
80. Aloia TA, Harpole DH Jr, Reed CE, Allegra C, Moore MB, Herndon JE 2nd, D'Amico TA: **Tumor marker expression is predictive of survival in patients with esophageal cancer.** *Ann Thorac Surg* 2001, **72:**859–866.
81. Berggvist AS, Bergqvist M, Brattstrom D, Hesselius P, Larsson A, Brodin O, Wagenius G: **Serum p53 autoantibodies as prognostic marker in patients with oesophageal carcinoma.** *Anticancer Res* 2001, **21:**4141–4145.
82. Makoto O, Takeda A, Ting-Leig L, Shinnichi O, Hisahiro M, Yutaka F, Yoshihiro N, Kobayashi S, Gunji Y, Suzuki T, Takenori O, *et al.*: **Prognostic significance of thymidine phosphorylase and p53 co-expression in esophageal squamous cell carcinoma.** *Oncol Rep* 2002, **9:**23–28.
83. Noguchi T, Takeno S, Shibata T, Uchida Y, Yokoyama S, Muller W: **Expression of heat shock protein 70 in grossly resected esophageal squamous cell carcinoma.** *Ann Thorac Surg* 2002, **74:**222–226.
84. Shimada H, Nabeya Y, Okazumi S, Matsubara H, Funami Y, Shiratori T, Hayashi H, Takeda A, Ochiai T: **Prognostic significance of serum p53 antibody in patients with esophageal squamous cell carcinoma.** *Surgery* 2002, **132:**41–47.
85. Takeno S, Noguchi T, Kikuchi R, Uchida Y, Yokoyama S, Muller W: **Prognostic value of cyclin B1 in patients with esophageal squamous cell carcinoma.** *Cancer* 2002, **94:**2874–2881.
86. Gibson MK, Abraham SC, Wu TT, Burtness B, Heitmiller RF, Heath E, Forastiere A: **Epidermal growth factor receptor, p53 mutation, and pathological response predict survival in patients with locally advanced esophageal cancer treated with preoperative chemoradiotherapy.** *Clin Canc Res Offic J Am Assoc Canc Res* 2003, **9:**6461–6468.
87. Ikeguchi M, Yamaguchi K, Kaibara N: **Survivin gene expression positively correlates with proliferative activity of cancer cells in esophageal cancer.** *Tumour Biol J Int Soc Oncodevelopmental Biol Med* 2003, **24:**40–45.
88. Kunisaki C, Imada T, Yamada R, Hatori S, Kinbara K, Watai K, Akiyama H, Nomura M, Matsuda G, Otsuka Y, Ono H, *et al.*: **Prognostic factors after chemoradiotherapy for patients with inoperable esophageal squamous cell carcinoma.** *Hepatogastroenterology* 2006, **53:**366–371.
89. Hsu PK, Li AF-Y, Wang Y-C, Hsieh C-C, Huang M-H, Hsu W-H, Hsu HS: **Reduced membranous beta-catenin protein expression is associated with metastasis and poor prognosis in squamous cell carcinoma of the esophagus.** *J Thorac Cardiovasc Surg* 2008, **135:**1029–1035.
90. Shimada H, Shiratori T, Takeda A, Matsushita K, Okazumi S, Akutsu Y, Matsubara H, Nomura F, Ochiai T: **Perioperative changes of serum p53 antibody titer is a predictor for survival in patients with esophageal squamous cell carcinoma.** *World J Surg* 2008, **33:**272–277.
91. Cheng T-H, Hsu P-K, Li AF-Y, Hung IC, Huang M-H, Hsu HS: **Correlation of p53, MDM2 and p14(ARF) protein expression in human esophageal squamous cell carcinoma.** *J Cancer Res Clin Oncol* 2009, **135:**1577–1582. Epub 2009 Jun 1572.
92. Okumura H, Kita Y, Yokomakura N, Uchikado Y, Setoyama T, Sakurai H, Omoto I, Matsumoto M, Owaki T, Ishigami S, Natsugoe S: **Nuclear expression of 14-3-3 sigma is related to prognosis in patients with esophageal squamous cell carcinoma.** *Anticancer Res* 2010, **30:**5175–5179.
93. Yamasaki M, Miyata H, Fujiwara Y, Takiguchi S, Nakajima K, Nishida T, Yasuda T, Matsuyama J, Mori M, Doki Y: **p53 genotype predicts response to chemotherapy in patients with squamous cell carcinoma of the esophagus.** *Ann Surg Oncol* 2010, **17:**634–642. Epub 2009 Nov 2026.
94. Yen C-C, Tsao Y-P, Chen PC-H, Wu Y-C, Liu J-H, Pan C-C, Liu C-Y, Tzeng C-H, Chen P-M, Chen Y-J, Lin C-H, *et al.*: **PML protein as a prognostic molecular marker for patients with esophageal squamous cell carcinomas receiving primary surgery.** *J Surg Oncol* 2011, **103:**761–767. 710.1002/jso.21855. Epub 22011 Jan 21815.
95. Blanchard P, Quero L, Pacault V, Schlageter MH, Baruch-Hennequin V, Hennequin C: **Prognostic significance of anti-p53 and anti-KRas circulating antibodies in esophageal cancer patients treated with chemoradiotherapy.** *BMC Canc* 2012, **12:**119.
96. Nakamura T, Nekarda H, Hoelscher AH, Bollschweiler E, Harbeck N, Becker K, Siewert JR, Harbec N: **Prognostic value of DNA ploidy and c-erbB-2 oncoprotein overexpression in adenocarcinoma of Barrett's esophagus.** *Cancer* 1994, **73:**1785–1794.
97. Mimura K, Kono K, Hanawa M, Mitsui F, Sugai H, Miyagawa N, Ooi A, Fujii H: **Frequencies of HER-2/neu expression and gene amplification in patients with oesophageal squamous cell carcinoma.** *Br J Cancer* 2005, **92:**1253–1260.
98. Dreilich M, Wanders A, Brattstrom D, Bergstrom S, Hesselius P, Wagenius G, Bergqvist M: **HER-2 overexpression (3+) in patients with squamous cell esophageal carcinoma correlates with poorer survival.** *Dis Esophagus* 2006, **19:**224–231.
99. Rauser S, Weis R, Braselmann H, Feith M, Stein HJ, Langer R, Hutzler P, Hausmann M, Lassmann S, Siewert JR, Hofler H, *et al.*: **Significance of HER2 low-level copy gain in Barrett's cancer: implications for fluorescence in situ hybridization testing in tissues.** *Clin Cancer Res* 2007, **13:**5115–5123.
100. Berg D, Wolff C, Langer R, Schuster T, Feith M, Slotta-Huspenina J, Malinowsky K, Becker KF: **Discovery of new molecular subtypes in oesophageal adenocarcinoma.** *PloS One* 2011, **6:**e23985. doi: 10.1371/journal.pone.0023985Epub 2011 Sep 23.
101. Kato H, Nakajima M, Masuda N, Faried A, Sohda M, Fukai Y, Miyazaki T, Fukuchi M, Tsukada K, Kuwano H: **Expression of RCAS1 in esophageal squamous cell carcinoma is associated with a poor prognosis.** *J Surg Oncol* 2005, **90:**89–94.
102. Falkenback D, Nilbert M, Oberg S, Johansson J: **Prognostic value of cell adhesion in esophageal adenocarcinomas.** *Dis Esophagus* 2008, **21:**97–102.
103. Zhao XJ, Li H, Chen H, Liu Y-X, Zhang L-H, Liu S-X, Feng QL: **Expression of e-cadherin and beta-catenin in human esophageal squamous cell carcinoma: relationships with prognosis.** *World J Gastroenterol* 2003, **9:**225–232.
104. Shimada Y, Hashimoto Y, Kan T, Kawamura J, Okumura T, Soma T, Kondo K, Teratani N, Watanabe G, Ino Y, Sakamoto M, *et al.*: **Prognostic significance of dysadherin expression in esophageal squamous cell carcinoma.** *Oncology* 2004. City.
105. Takeno S, Noguchi T, Fumoto S, Kimura Y, Shibata T, Kawahara K: **E-cadherin expression in patients with esophageal squamous cell carcinoma: promoter hypermethylation, Snail overexpression, and clinicopathologic implications.** *Am J Clin Pathol* 2004, **122:**78–84.
106. Natsugoe S, Uchikado Y, Okumura H, Matsumoto M, Setoyama T, Tamotsu K, Kita Y, Sakamoto A, Owaki T, Ishigami S, Aikou T: **Snail plays a key role in E-cadherin-preserved esophageal squamous cell carcinoma.** *Oncol Rep* 2007, **17:**517–523.
107. Setoyama T, Natsugoe S, Okumura H, Matsumoto M, Uchikado Y, Yokomakura N, Ishigami S, Aikou T: **alpha-catenin is a significant prognostic factor than E-cadherin in esophageal squamous cell carcinoma.** *J Surg Oncol* 2007, **95:**148–155.
108. Sasaki K, Natsugoe S, Ishigami S, Matsumoto M, Okumura H, Setoyama T, Uchikado Y, Kita Y, Tamotsu K, Sakamoto A, Owaki T, *et al.*: **Significance of Twist expression and its association with E-cadherin in esophageal squamous cell carcinoma.** *J Exp Clin Cancer Res* 2009, **28:**158.
109. Chung Y, Law S, Kwong DL, Luk JM: **Serum soluble E-cadherin is a potential prognostic marker in esophageal squamous cell carcinoma.** *Dis Esophagus* 2011, **24:**49–55. 10.1111/j.1442-2050.2010.01093.x. Epub 02010 Aug 01030.
110. Nozoe T, Saeki H, Sugimachi K: **Significance of preoperative elevation of serum C-reactive protein as an indicator of prognosis in esophageal carcinoma.** *Am J Surg* 2001, **182:**197–201.
111. Ikeda M, Natsugoe S, Ueno S, Baba M, Aikou T: **Significant host- and tumor-related factors for predicting prognosis in patients with esophageal carcinoma.** *Ann Surg* 2003, **238:**197–202.
112. Nozoe T, Korenaga D, Futatsugi M, Saeki H, Maehara Y, Sugimachi K: **Immunohistochemical expression of C-reactive protein in squamous cell carcinoma of the esophagus - significance as a tumor marker.** *Cancer Lett* 2003, **192:**89–95.
113. Shimada H, Nabeya Y, Okazumi S, Matsubara H, Shiratori T, Aoki T, Sugaya M, Miyazawa Y, Hayashi H, Miyazaki S, Ochiai T: **Elevation of preoperative serum C-reactive protein level is related to poor prognosis in esophageal squamous cell carcinoma.** *J Surg Oncol* 2003, **83:**248–252.
114. Gockel I, Dirksen K, Messow C-M, Junginger T: **Significance of preoperative C-reactive protein as a parameter of the perioperative course and long-term prognosis in squamous cell carcinoma and adenocarcinoma of the oesophagus.** *World J Gastroenterol* 2006, **12:**3746–3750.
115. Wang C-Y, Hsieh M-J, Chiu Y-C, Li S-H, Huang H-W, Fang F-M, Huang YJ: **Higher serum C-reactive protein concentration and hypoalbuminemia are poor prognostic indicators in patients with esophageal cancer undergoing radiotherapy.** *Radiother Oncol* 2009, **92:**270–275. Epub 2009 Feb 2003.
116. Zingg U, Forberger J, Rajcic B, Langton C, Jamieson GG: **Association of C-reactive protein levels and long-term survival after neoadjuvant therapy and esophagectomy for esophageal cancer.** *J Gastrointest Surg* 2010, **14:**462–469. Epub 2009 Nov 2025.
117. Nakatsu T, Motoyama S, Fa Maruyama K, Usami S, Sato Y, Miura M, Hinai Y, Saito H, Minamiya Y, Murata K, Ogawa J: **Tumoral CRP expression in thoracic esophageal squamous cell cancers is associated with poor outcomes.** *Surg Today* 2012, **42:**652–658. Epub 2012 Feb 2021.
118. Shimada H, Nabeya Y, Okazumi S, Matsubara H, Shiratori T, Gunji Y, Kobayashi S, Hayashi H, Ochiai T: **Prediction of survival with squamous cell carcinoma antigen in patients with resectable esophageal squamous cell carcinoma.** *Surgery* 2003, **133:**486–494.
119. Kosugi S, Nishimaki T, Kanda T, Nakagawa S, Ohashi M, Hatakeyama K: **Clinical significance of serum carcinoembryonic antigen, carbohydrate antigen 19–9, and squamous cell carcinoma antigen levels in esophageal cancer patients.** *World J Surg* 2004, **28:**680–685.
120. Shimada Y, Watanabe G, Kawamura J, Soma T, Okabe M, Ito T, Inoue H, Kondo M, Mori Y, Tanaka E, Imamura M: **Clinical significance of osteopontin in esophageal squamous cell carcinoma: comparison with common tumor markers.** *Oncology* 2005, **68:**285–292.
121. Cao M, Yie SM, Wu SM, Chen S, Lou B, He X, Ye SR, Xie K, Rao L, Gao E, Ye NY: **Detection of survivin-expressing circulating cancer cells in the peripheral blood of patients with esophageal squamous cell carcinoma and its clinical significance.** *Clin Exp Metastasis* 2009, **26:**751–758.
122. Brattstrom D, Wagenius G, FSandstrom P, Dreilich M, Bergstrom S, Goike H, Hesselius P, Bergqvist M: **Newly developed assay measuring cytokeratins 8, 18 and 19 in serum is correlated to survival and tumor volume in patients with esophageal carcinoma.** *Dis Esophagus* 2005, **18:**298–303.
123. Valencia Julve J, Alonso Orduna V, Esco Baron R, Lopez-Mata M, Mendez Villamon A: **Influence of hemoglobin levels on survival after radical treatment of esophageal carcinoma with radiotherapy.** *Clin Translational Oncol Offic Publ Federation Spanish Oncol Soc Nat Canc Inst Mexico* 2006, **8:**22–30.
124. Zhao KL, Liu G, Jiang GL, Wang Y, Zhong LJ, Yao WQ, Guo XM, Wu GD, Zhu LX, Shi XH: **Association of haemoglobin level with morbidity and mortality of patients with locally advanced oesophageal carcinoma undergoing radiotherapy--a secondary analysis of three consecutive clinical phase III trials.** *Clin Oncol (R Coll Radiol)* 2006, **18:**621–627.
125. Rades D, Golke H, Schild SE, Kilic E: **Impact of VEGF and VEGF receptor 1 (FLT1) expression on the prognosis of stage III esophageal cancer patients after radiochemotherapy.** *Strahlenther Onkol Organ Deutschen Rontgengesellschaft [et al.]* 2008, **184:**416–420.
126. Zenda S, Hironaka S, Boku N, Yamazaki K, Yasui H, Fukutomi A, Yoshino T, Onozawa Y, Nishimura T: **Impact of hemoglobin level on survival in definitive chemoradiotherapy for T4/M1 lymph node esophageal cancer.** *Dis Esophagus Offic J Int Soc Dis Esophagus / ISDE* 2008, **21:**195–200.
127. Gould Rothberg BE, Bracken MB, Rimm DL: **Tissue biomarkers for prognosis in cutaneous melanoma: a systematic review and meta-analysis.** *J Natl Canc Inst* 2009, **101:**452–474.
128. Hemingway H, Philipson P, Chen R, Fitzpatrick NK, Damant J, Shipley M, Abrams KR, Moreno S, McAllister KS, Palmer S, Kaski JC, *et al.*: **Evaluating the quality of research into a single prognostic biomarker: a systematic review and meta-analysis of 83 studies of C-reactive protein in stable coronary artery disease.** *PloS Med* 2010, **7:**e1000286. doi: 0.1371/journal.pmed.1000286.
129. Mrena J, Wiksten J-P, Kokkola A, Nordling S, Ristimaki A, Haglund C: **COX-2 is associated with proliferation and apoptosis markers and serves as an independent prognostic factor in gastric cancer.** *Tumour Biol* 2010, **31:**1–7. 10.1007/s13277-13009-10001-13274. Epub 12009 Dec 13218.
130. Chen J, Li T, Wu Y, He L, Zhang L, Shi T, Yi Z, Liu M, Pang X: **Prognostic significance of vascular endothelial growth factor expression in gastric carcinoma: a meta-analysis.** *J Canc Res Clin Oncol* 2011, **137:**1799–1812.
131. Des Guetz G, Uzzan B, Nicolas P, Cucherat M, Morere JF, Benamouzig R, Breau JL, Perret GY: **Microvessel density and VEGF expression are prognostic factors in colorectal cancer. Meta-analysis of the literature.** *Br J Canc* 2006, **94:**1823–1832.
132. Altieri DC: **Survivin, cancer networks and pathway-directed drug discovery.** *Nat Rev Canc* 2008, **8:**61–70.
133. Lee MH, Yang HY: **Negative regulators of cyclin-dependent kinases and their roles in cancers.** *Cell Mol life Sci: CMLS* 2001, **58:**1907–1922.
134. Chu IM, Hengst L, Slingerland JM: **The Cdk inhibitor p27 in human cancer: prognostic potential and relevance to anticancer therapy.** *Nat Rev Canc* 2008, **8:**253–267.
135. Al-Maghrabi J, Al-Ahwal M, Buhmeida A, Syrjanen K, Sibyani A, Emam E, Ghanim A, Al-Qahtani M: **Expression of cell cycle regulators p21 and p27 as predictors of disease outcome in colorectal carcinoma.** *J Gastrointest Canc* 2012, **43:**279–287.
136. Hugo H, Ackland ML, Blick T, Lawrence MG, Clements JA, Williams ED, Thompson EW: **Epithelial–mesenchymal and mesenchymal–epithelial transitions in carcinoma progression.** *J Cell Physiol* 2007, **213:**374–383.
137. Hirohashi S, Kanai Y: **Cell adhesion system and human cancer morphogenesis.** *Canc Sci* 2005, **94:**575–581.
138. Gaarenstroom KN, Kenter GG, Bonfrer JMG, Korse CM, Van de Vijver MJ, Fleuren GJ, Trimbos JB: **Can initial serum cyfra 21–1, SCC antigen, and TPA levels in squamous cell cervical cancer predict lymph node metastases or prognosis?** *Gynecol Oncol* 2000, **77:**164–170.
139. Hatzakis KD, Froudarakis ME, Bouros D, Tzanakis N, Karkavitsas N, Siafakas NM: **Prognostic value of serum tumor markers in patients with lung cancer.** *Respiration* 2002, **69:**25–29.
140. Mahmoud FA, Rivera NI: **The role of C-reactive protein as a prognostic indicator in advanced cancer.** *Curr Oncol Rep* 2002, **4:**250–255.
141. Begg CB, Mazumdar M: **Operating characteristics of a rank correlation test for publication bias.** *Biometrics* 1994, **50:**1088–1101.
