# Supplementary material for: Systematic review and meta-analysis of tumor biomarkers in predicting prognosis in esophageal cancer
Source: BMC Cancer. 2013 Nov 11;13:539. doi: 10.1186/1471-2407-13-539 (PMC3828582; doi:10.1186/1471-2407-13-539)
Supplement: Additional file 1: Table S1 — Definitions of 16 items of study reporting quality. [file 1471-2407-13-539-S1.doc]

| Supplementary Table S1. Definitions of 16 items of study reporting quality |
| --- |
| Study Design   1. Objectives or pre-specified hypothesis   State the study objectives, protocol or pre-specified hypothesis   1. Sample size   State a statistical sample size or power calculation   1. Follow-up description   State the follow-up period or the median follow-up time   1. Patients source   State healthcare setting from which patients were recruited   1. Patients selection criteria   State inclusion or exclusion criteria   1. Population characteristics   State the patients characteristics (e.g. age, gender and disease stage)   1. Flow of patients   State the number of patients included in each stage of the analysis and reason for dropout |
| Biomarker Mesurement   1. Sample handling   State the method of storage   1. Assay method   State the type of assay method used to measure biomarker   1. Manufacturer   State the name of company which makes the assay for biomarker or provide reagents or kits.   1. Cutpoint   State methods used to determine cutpoint. |
| Outcomes   1. Confounders   State the conventional risk factors (e.g. age, gender, deep of tumor, lymph node metastasis) or other biomarkers relating with the disease   1. Clinical endpoint   State the clinical endpoint |
| 1. Validation   State the outcome events checked by independent source (e.g. medical records, outpatient visits, by letter and by telephone) |
| Analysis   1. Multivaraite estimate   Adjusted for confound factors list above.   1. Missing value   State the number of patients with missing value for biomarker or confounders and how to deal with it. |
