# Supplementary material for: Systematic review and meta-analysis of tumor biomarkers in predicting prognosis in esophageal cancer
Source: BMC Cancer. 2013 Nov 11;13:539. doi: 10.1186/1471-2407-13-539 (PMC3828582; doi:10.1186/1471-2407-13-539)
Supplement: Additional file 2: Table S2 — The characteristics of the eligible studies for this systematic review. [file 1471-2407-13-539-S2.docx]

|  | Supplementary Table S2. The characteristics of the eligible studies for this systematic review. | | | | | | | | | | | | | | |
| --- | --- | --- | --- | --- | --- | --- | --- | --- | --- | --- | --- | --- | --- | --- | --- |
| Molecule | | First Author | Year | Country | Histology | Sample size | Mean/Median age | Male % | Tumor stage (Ⅰ+Ⅱ) % | Test Method | cutoff value | Abnormal expression % | HR | ll | ul |
| Angiogenesis | |  |  |  |  |  |  |  |  |  |  |  |  |  |  |
| COX-2 | | Anna Sivula | 2005 | Finland | ESCC | 36 | 58.8 | 52.8 | 52.8 | IHC | Stain score >=2 | 30.6 | 0.13 | 0.03 | 0.63 |
|  | | Tadahiro Nozoe | 2005 | Japan | ESCC | 76 | 65 | 86.8 | 80.3 | IHC | 50% cell positive | 36.8 | 1.24 | 0.45 | 3.43 |
|  | | Pradeep Bhandari | 2006 | UK | EADC | 90 | 65 | 90 | NR | IHC | Median score >=200 | 50 | 2.91 | 1.78 | 4.76 |
|  | | Mee Sun Yoon | 2011 | Korea | ESCC | 44 | 64 | 97.7 | 29.5 | IHC | Intensity of 2 or 3 and 10% stained or intensity 1 and >50% stained | 38.6 | 2.34 | 1.11 | 4.91 |
|  | | Christianne J. | 2012 | Finland | EADC | 145 | 65.7 | 82.8 | 55.9 | IHC | Stain score >=2 | 79.3 | 3.50 | 1.60 | 7.90 |
|  | | J.-X. Huang | 2012 | China | ESCC | 78 | 58.1 | 87.2 | 55.1 | IHC | Cytoplasm and/or nuclear staining | 55.1 | 1.11 | 0.53 | 2.32 |
| VEGF | | S Uchida | 1998 | Japan | EC | 109 | 63.5 | 81.7 | 56 | IHC | 10% cell positive | 59.6 | 1.80 | 0.75 | 5.01 |
|  | | Y Shimada | 1999 | Japan | ESCC | 116 | NR | 84.5 | 33.6 | IHC | 10% cell positive | 69 | 1.59 | 0.69 | 4.04 |
|  | | Chih-H | 2000 | Japan | ESCC | 117 | NR | 90.6 | 52.8 | IHC | 80% cell positive | 30.8 | 3.35 | 1.02 | 10.98 |
|  | | Hideaki | 2001 | Japan | ESCC | 82 | 65 | 85.4 | 43.9 | ELISA | Median | 36.6 | 3.83 | 1.82 | 8.08 |
|  | | Myung-Ju | 2002 | Korea | ESCC | 81 | 60 | 93.8 | 43.2 | IHC | 30% cell positive | 51.3 | 1.27 | 0.61 | 2.63 |
|  | | H Shimada | 2002 | Japan | ESCC | 52 | 65 | 83 | 19.2 | IHC | 10% cell positive | 44.2 | 2.43 | 1.19 | 4.94 |
|  | | Hiroyuki | 2002 | Japan | ESCC | 64 | 61.4 | 85.9 | 59.4 | IHC | 80% cell positive | 37.5 | 1.03 | 0.46 | 2.34 |
|  | | A.R Rosa | 2003 | Brazil | ESCC | 47 | 55 | 87.2 | 48.9 | IHC | 30% cell positive | 40.4 | 0.48 | 0.18 | 1.32 |
|  | | Yutaka | 2003 | Japan | ESCC | 92 | 60.2 | 91.3 | 50 | IHC | 10% cell positive | 23.9 | 2.40 | 1.10 | 5.34 |
|  | | Martin | 2005 | Sweden | ESCC | 42 | NR | 73.8 | NR | ELISA | Median | 48 | 1.00 | 1.00 | 1.00 |
|  | | Joon Yong | 2006 | Korea | ESCC | 51 | NR | 92.2 | 54.9 | IHC | 10% cell positive | 58.8 | 7.21 | 1.71 | 30.4 |
|  | | Takayuki | 2007 | Japan | ESCC | 51 | 68 | 82 | 35 | IHC | Strong staining | 31 | 0.90 | 0.42 | 1.96 |
|  | | Ching Tzao | 2008 | Taiwan | ESCC | 85 | NR | 94.1 | 56.5 | IHC | 10% cell positive | 65.9 | 1.73 | 1.04 | 2.92 |
|  | | Akemi | 2008 | Japan | ESCC | 81 | NR | 95.1 | NR | IHC | 10% cell positive | 87 | 1.69 | 0.65 | 4.41 |
|  | | Pengfei Liu | 2009 | China | ESCC | 73 | 61 | 76.7 | 50.7 | IHC | 30% cell positive | 53.4 | 2.23 | 1.35 | 5.00 |
|  | | Leandro | 2009 | Brazil | EADC | 38 | 60.6 | 78.9 | 36.8 | IHC | 30% cell positive | 50 | 0.37 | 0.10 | 1.44 |
|  | | Miroslaw | 2010 | Poland | ESCC | 149 | 62 | 91.9 | 31.5 | ELISA | Median | 41.7 | 2.18 | 1.37 | 3.47 |
|  | | Zhi-Gong Sun | 2010 | China | ESCC | 82 | NR | 78 | NR | RT-PCR | Median | 51.2 | 2.51 | 1.21 | 5.19 |
|  | | Tatsuya | 2010 | Japan | ESCC | 106 | NR | 82.1 | 39.6 | RT-PCR | Median | 50 | 1.64 | 0.97 | 2.78 |
| Evading apoptosis | |  |  |  |  |  |  |  |  |  |  |  |  |  |  |
| survivin | | S. Mega | 2006 | Japan | ESCC | 122 | 62.3 | 86.1 | 61.5 | IHC | Stain score <1 | 55.7 | 0.69 | 0.37 | 1.28 |
|  | | Antonio Rosato | 2006 | Italy | ESCC | 56 | 62 | 87.5 | 41.1 | IHC | 20% cell positive | 51.8 | 2.40 | 1.20 | 4.80 |
|  | | K.-F. Hsu | 2009 | China | ESCC | 46 | 61.4 | 93.5 | 63.0 | IHC | Stain score >=175 | 50 | 2.76 | 1.28 | 5.91 |
|  | | Shinuke Takeno | 2010 | Japan | ESCC | 71 | 63.8 | 88.7 | 52.1 | IHC | 10% cell positive | 31 | 1.49 | 1.04 | 2.13 |
|  | | Andreas-Cloudius Hoffmann | 2010 | Germany | EC | 62 | 61 | 85.5 |  | PCR | Medain | 77 | 6.60 | 1.97 | 22.12 |
| Insensitivity to antigrowth signals | |  |  |  |  |  |  |  |  |  |  |  |  |  |  |
| p21 | | M Sarbia | 1998 | Germany | ESCC | 172 | 58 | 77.3 | NR | IHC | 50% cell positive | 16.8 | 1.80 | 1.13 | 2.88 |
|  | | Shoji Natsugoe | 1999 | Japan | EC | 111 | 64 | 92.8 | 29.7 | IHC | 50% cell positive | 43.2 | 0.54 | 0.30 | 0.97 |
|  | | Marcelo E. Nita | 1999 | Japan | ESCC | 62 | 62.9 | 80.6 | 67.7 | IHC | Median stain score | 46.8 | 0.65 | 0.43 | 0.96 |
|  | | Toshihiro Hirai | 1999 | Japan | EC | 64 | NR | 92.2 | 40.6 | IHC | 10% cell positive | 42.2 | 3.41 | 1.39 | 8.37 |
|  | | Y Shimada | 1999 | Japan | ESCC | 116 | 63.9 | 84.5 | 33.6 | IHC | 50% cell positive | 8.6 | 3.95 | 0.43 | 30.86 |
|  | | T. Wakamura | 2004 | Japan | ESCC | 76 | NR | NR | NR | IHC | 10% cell positive | 30 | 0.42 | 0.30 | 0.59 |
|  | | Manabu Matsumoto | 2004 | Japan | ESCC | 137 | 62.4 | 88.3 | 67.2 | IHC | 5% cell positive | 40.9 | 1.77 | 0.90 | 3.49 |
|  | | Yih-Gang Goan | 2005 | China | ESCC | 40 | NR | 95 | 47.5 | IHC | 50% cell positive | 50 | 2.30 | 1.06 | 4.99 |
|  | | Noushin Taghavi | 2010 | Iran | ESCC | 80 | 61.4 | 56.3 | NR | IHC | 50% cell positive | 37.5 | 1.82 | 1.02 | 3.25 |
| p27 | | Takashi Anayama | 1998 | Japan | ESCC | 77 | 62 | 88.3 | 50.6 | IHC | 50% cell positive | 67.5 | 0.47 | 0.25 | 0.98 |
|  | | Atsushi Itami | 1999 | Japan | EC | 128 | 63.9 | 81.3 | 50.8 | IHC | 50% cell positive | 50 | 0.75 | 0.41 | 1.38 |
|  | | M. Yasunaya | 1999 | Japan | ESCC | 40 | 66.4 | 92.5 | 55 | IHC | 10% cell positive | 62.5 | 2.83 | 1.10 | 9.71 |
|  | | Hitoshi Shiozaki | 2000 | Japan | ESCC | 77 | NR | NR | 27.3 | IHC | 10% cell positive | 40.2 | 3.93 | 1.31 | 11.78 |
|  | | Awad Shamma | 2000 | Japan | ESCC | 106 | 62.5 | 82.1 | 65.1 | IHC | 10% cell positive | 61.3 | 2.28 | 1.18 | 4.44 |
|  | | K Nishioka | 2001 | Japna | ESCC | 100 | 61.2 | 80 | 43 | IHC | NR | 38.1 | 2.12 | 0.85 | 5.32 |
|  | | J.-X. Huang | 2012 | China | ESCC | 78 | 58.1 | 87.2 | 55.1 | IHC | 50% cell positive | 57.7 | 2.99 | 1.39 | 6.47 |
| Limitless replicative potential | |  |  |  |  |  |  |  |  |  |  |  |  |  |  |
| cyclin D1 | | Takashi Anayama | 1998 | Japan | ESCC | 77 | 62 | 88.3 | 50.6 | IHC | No strain | 27.3 | 2.44 | 1.29 | 5.69 |
|  | | Mario Sarbia | 1999 | Germany | ESCC | 172 | 58 | 77.3 | NR | IHC | No stain | 72.7 | 2.14 | 1.34 | 3.42 |
|  | | Atsushi Itami | 1999 | Japan | EC | 156 | 63.7 | 82.1 | 51.9 | IHC | 10% cell positive | 32.7 | 2.20 | 1.15 | 4.21 |
|  | | Toshihiro Hirai | 1999 | Japan | EC | 64 | NR | 92.2 | 40.6 | IHC | 10% cell positive | 43.8 | 0.41 | 0.16 | 0.99 |
|  | | Y Shimada | 1999 | Japan | ESCC | 116 | 63.9 | 84.5 | 33.6 | IHC | 10% cell positive | 39.7 | 1.84 | 0.76 | 4.47 |
|  | | Hitoshi Shiozaki | 2000 | Japan | ESCC | 77 | NR | NR | 27.3 | IHC | Normal epithelium | 46.7 | 1.16 | 0.53 | 2.53 |
|  | | Masahide Ikeguchi | 2001 | Japan | ESCC | 148 | 66 | 90 | 54.7 | IHC | 20% cell positive | 40.5 | 1.46 | 0.9 | 2.38 |
|  | | Shigenao Nagasawa | 2001 | Japan | ESCC | 86 | 63 | 88.4 | 51.2 | IHC | 5% cell positive | 26.7 | 2.47 | 1.07 | 5.72 |
|  | | Imamura | 2001 | Japan | EC | 416 | 62.4 | 87.5 | 62.3 | IHC | 10% cell positive | 39.9 | 1.42 | 1.04 | 1.94 |
|  | | M. Shinohara | 2002 | Japan | ESCC | 114 | 60 | 88.2 | 36.8 | IHC | 20% cell positive | 41.2 | 0.70 | 0.40 | 1.23 |
|  | | Dilek Guner | 2003 | Germany | ESCC | 53 | 54.9 | 75.5 | 62.3 | IHC | 60% cell positive | 82.7 | 2.70 | 1.28 | 5.71 |
|  | | Manabu Matsumoto | 2004 | Japan | ESCC | 137 | 62.4 | 88.3 | 67.2 | IHC | 5% cell positive | 30.7 | 3.05 | 1.47 | 6.29 |
|  | | Minoru Fukuchi | 2006 | Japan | ESCC | 119 | 62.2 | 86.6 | 61.3 | IHC | 10% cell positive | 52.1 | 2.04 | 1.08 | 3.84 |
|  | | H Takeshita | 2010 | Japan | ESCC | 96 | 64.2 | 77.1 | 69.8 | PCR | Plasma CCND/DRD2 ratio > 1.33 | 43.7 | 5.99 | 1.23 | 29.41 |
|  | | M.-T. Wang | 2012 | China | ESCC | 100 | 59 | 78 | 48 | PCR | Mean+2SD | 41.0 | 2.26 | 1.15 | 4.44 |
| HER-2 | | Tsutomu | 1994 | Germany | EADC | 80 | 60.4 | 91.3 | 41.3 | IHC | Stain score >2 | 18.8 | 4.10 | 1.40 | 11.8 |
|  | | K Mimura | 2005 | Japan | ESCC | 66 | 65.3 | 93.9 | 59.1 | IHC | Stain score >=1 | 30.3 | 0.92 | 0.35 | 2.41 |
|  | | M. Dreilich | 2006 | Saeden | EC | 97 | NR | 71.1 | NR | IHC | Stain score >3 | 17.5 | 1.14 | 0.68 | 1.60 |
|  | | Sandra Rauser | 2007 | Germany | EADC | 124 | 65.6 | NR | NR | FISH | NR | NR | 1.80 | 0.90 | 3.6 |
|  | | Deniela Bery | 2011 | Germany | EADC | 87 | NR | NR | NR | RPPA | > 1276 MVS | 19.5 | 1.97 | 1.01 | 3.82 |
|  | | Harry Y. Yoon | 2012 | USA | EC | 708 | 63.8 | NR | NR | IHC | Faint intensity in 10% or more | 16.8 | 0.84 | 0.7 | 1.14 |
| Ki-67 | | Y Shimada | 1999 | Japan | ESCC | 116 | 63.9 | 84.5 | 33.6 | IHC | 30% cell positive | 41.4 | 0.81 | 0.66 | 3.60 |
|  | | Masahide Ikeguchi | 2003 | Japan | EC | 57 | 65.2 | 94.7 | 45.6 | IHC | Mean value of Ki-67 LI | | 1.03 | 0.49 | 2.17 |
|  | | Hiroyuki Kato | 2005 | Japan | ESCC | 114 | 62.2 | 85.1 | 59.6 | IHC | NR | NR | 1.39 | 0.77 | 2.54 |
|  | | D. Falkenback | 2008 | Sweden | EADC | 59 | 66 | 86.4 | 62 | IHC | 10% cell positive | 85 | 0.26 | 0.11 | 0.59 |
|  | | J.-X. Huang | 2012 | China | ESCC | 78 | 58.1 | 87.2 | 55.1 | IHC | 10% cell positive | 73 | 0.68 | 0.13 | 3.65 |
| p53 | | Apinop | 1995 | Canada | ESCC | 80 | 63 | 76.3 | 37.5 | IHC | NO stain | 50 | 1.40 | 1.10 | 1.90 |
|  | | A G Casson | 1998 | UK | EC | 132 | 64.3 | 90.9 | NR | IHC | 25% cell positive | 47.7 | 1.73 | 1.17 | 2.57 |
|  | | J. Pomp | 1998 | Netherlands | EC | 69 | NR | 79.7 | 69.6 | IHC | 20% cell positive | 53.6 | 1.96 | 1.15 | 3.31 |
|  | | Akira | 1999 | Japan | ESCC | 239 | 62.4 | 90.4 | 67.4 | IHC | No stain | 48.1 | 1.02 | 0.64 | 1.61 |
|  | | Toshihiro | 1999 | Japan | EC | 64 | NR | 92.2 | 40.6 | IHC | 10% cell positive | 48.4 | 0.58 | 0.26 | 1.30 |
|  | | M. Kuwahara | 1999 | Japan | EC | 64 | NR | NR | 40.6 | IHC | 10% cell positive | 48.4 | 1.73 | 0.77 | 3.90 |
|  | | Y Shimada | 1999 | Japan | EC | 116 | 63.9 | 84.5 | 33.6 | IHC | 10% cell positive | 43.1 | 1.27 | 0.53 | 3.01 |
|  | | Masahide | 2000 | Japan | ESCC | 191 | 63 | 89.5 | 51.8 | IHC | 50% cell positive | 41 | 0.74 | 0.48 | 1.15 |
|  | | Pau M. | 2000 | Germany | EADC | 59 | 62.7 | 91.5 | 67.8 | PCR-SSCP | p53 exons show | 50.8 | 2.47 | 1.13 | 5.34 |
|  | | Thomas | 2001 | USA | EC | 61 | 62 | 82 | NR | IHC | Median score | 32.8 | 1.60 | 1.02 | 2.51 |
|  | | Ann-Sofi | 2001 | Sweden | EC | 34 | NR | 62 | NR | ELISA | E450 less than the low control | 21 | 2.77 | 1.01 | 7.57 |
|  | | Tsuyoshi | 2002 | Germany | ESCC | 71 | 63.8 | 88.7 | 52.1 | IHC | 10% cell positive | 63.4 | 1.10 | 0.76 | 1.58 |
|  | | Oota Makoto | 2002 | Japna | ESCC | 96 | 65 | 86.5 | 58.3 | IHC | 10% cell positive | 45.8 | 1.39 | 0.73 | 2.62 |
|  | | H Shimada | 2002 | Japan | ESCC | 52 | 65 | 83 | NR | IHC | 10% cell positive | 57.7 | 1.74 | 0.87 | 3.74 |
|  | | Hideaki | 2002 | Japan | ESCC | 105 | 65 | 88 | 70.5 | ELISA | 1.3U/ml | 28.6 | 2.14 | 0.92 | 4.98 |
|  | | M Shinohara | 2002 | Japan | ESCC | 114 | NR | 88.7 | NR | IHC | 5% cell positive | 71.1 | 0.87 | 0.44 | 1.70 |
|  | | Shinsuke | 2002 | Germany | ESCC | 71 | 63.8 | 88.7 | 59.2 | IHC | 10% cell positive | 63.4 | 0.83 | 0.43 | 1.67 |
|  | | Myung-Ju | 2002 | Korea | ESCC | 79 | 60 | 93.7 | 41.8 | IHC | 10% cell positive | 51.9 | 0.58 | 0.27 | 1.23 |
|  | | Michael K. | 2003 | USA | EC | 54 | 57.5 | 83.3 | NR | PCR-SSCP | p53 exons show | 63 | 0.45 | 0.19 | 1.10 |
|  | | A.R. Rosa | 2003 | Brazil | ESCC | 47 | NR | 87.2 | 48.9 | IHC | 10% cell positive | 53.2 | 0.76 | 0.27 | 2.13 |
|  | | Masahide | 2003 | Japan | ESCC | 75 | 67 | 94.7 | 45.6 | IHC | Median stain | 50.1 | 0.58 | 0.25 | 1.32 |
|  | | Manabu | 2004 | Japan | ESCC | 137 | 62.4 | 88.3 | 67.2 | IHC | 5% cell positive | 65.7 | 2.41 | 0.97 | 5.99 |
|  | | Chikara | 2006 | Japan | ESCC | 41 | 64.4 | 97.6 | NR | PCR-SSCP | p53 exons show | 61 | 3.82 | 1.35 | 10.85 |
|  | | Akemi | 2008 | Japan | ESCC | 81 | NR | NR | NR | IHC | 10% cell positive | 60 | 0.90 | 0.44 | 1.84 |
|  | | Po-Kuei Hsu | 2008 | Taiwan | ESCC | 68 | 65 | 97.1 | NR | IHC | 25% cell positive | 63.2 | 1.20 | 0.09 | 2.07 |
|  | | Hideaki | 2008 | Japan | ESCC | 110 | 65 | 88 | 59.1 | ELISA | 1.3U/ml | 31.8 | 3.04 | 1.11 | 8.33 |
|  | | Tzu-Hao Cheng | 2009 | Taiwan | ESCC | 119 | 64.9 | 95.8 | NR | IHC | 25% cell positive | 51.3 | 0.15 | 0.50 | 1.64 |
|  | | Leadro Totti | 2009 | Brazil | EADC | 38 | 60.6 | 78.9 | 36.8 | IHC | 10% cell positive | 52.6 | 1.43 | 0.43 | 4.73 |
|  | | Hiroshi | 2010 | Japan | ESCC | 248 | 63.8 | 92.7 | 47.6 | IHC | 10% cell positive | 51.6 | 1.50 | 1.10 | 1.90 |
|  | | Makoto | 2010 | Japan | ESCC | 97 | 61.9 | 86.6 | 11.3 | PCR-SSCP | p53 exons show | 47.4 | 1.68 | 0.96 | 2.91 |
|  | | Chueh-Chuan | 2011 | Taiwan | ESCC | 61 | NR | 82 | 44.3 | PCR-SSCP | p53 exons show | 59 | 3.12 | 1.21 | 8.05 |
|  | | Pierre | 2012 | France | EC | 97 | 60 | 84 | NR | ELISA | Median | 29 | 1.80 | 1.03 | 2.90 |
| Tissue invasion and metastasis | |  |  |  |  |  |  |  |  |  |  |  |  |  |  |
| E-cadherin | | Y Shimada | 1999 | Japan | ESCC | 116 | 63.9 | 84.5 | 33.6 | IHC | 90% cell positive | 81 | 3.38 | 1.16 | 11.1 |
|  | | Imamura | 2001 | Japan | EC | 416 | 62.4 | 87.5 | 62.3 | IHC | Stain score<4 | 57.7 | 1.41 | 1.01 | 1.96 |
|  | | Xi-Jiang Zhao | 2003 | China | ESCC | 106 | 59 | 71.7 | 40.6 | IHC | 10% cell positive | 20.8 | 1.54 | 1.08 | 2.19 |
|  | | Yutaka Shimada | 2004 | Japan | EC | 117 | 63.4 | 88 | 55.6 | IHC | 50% cell positive | 65 | 1.39 | 0.73 | 2.78 |
|  | | Shinsuke Takeno | 2004 | Japan | ESCC | 93 | 64 | 84.9 | 50.5 | IHC | 50% cell positive | 59.1 | 1.47 | 1.04 | 2.13 |
|  | | Shoji Natsugoe | 2007 | Japan | ESCC | 194 | 63.6 | 90.7 | 44.8 | IHC | Normal epithelium | 58.8 | 1.03 | 0.68 | 1.55 |
|  | | Tetsuro Setoyama | 2007 | Japan | ESCC | 205 | 64 | 89.3 | 56.6 | IHC | Normal epithelium | 52.2 | 0.77 | 0.56 | 1.07 |
|  | | D. Falkenback | 2008 | Sweden | EADC | 59 | 66 | 86.4 | 62 | IHC | Reduced membranous stainnig | 75 | 3.30 | 1.00 | 11.1 |
|  | | Ken Sasaki | 2009 | Japan | ESCC | 166 | 64.3 | 89.8 | 48.2 | IHC | Normal epithelium | 59.6 | 2.25 | 1.08 | 4.42 |
|  | | Y. Chung | 2011 | Singapore | ESCC | 97 | 64.1 | 79.4 | 50.5 | ELISA | Median | 50 | 1.10 | 1.03 | 1.19 |
| Serum markers | |  |  |  |  |  |  |  |  |  |  |  |  |  |  |
| CRP | | Tadahiro Nozoe | 2001 | Japan | EC | 262 | 62.4 | 86.6 | 59.9 | LPIA | 5 mg/L | 32.1 | 3.30 | 2.17 | 5.00 |
|  | | Tadahiro Nozoe | 2003 | Japan | ESCC | 37 | 66 | 86.5 | 51.4 | IHC | 10% cell positive | 48.6 | 3.27 | 1.23 | 8.70 |
|  | | Masanori Ikeda | 2003 | Japan | EC | 356 | 65 | 91.9 | 52.2 | ELISA | 0.5 mg/dl | 41.9 | 1.52 | 1.05 | 2.21 |
|  | | Hideaki Shimada | 2003 | Japan | ESCC | 150 | 65 | 85 | 46.7 | ELISA | 1.0 mg/dl | 23.3 | 1.42 | 0.83 | 2.41 |
|  | | Ines Gockel | 2006 | Germany | EC | 291 | 59 | 82.5 | NR | LEHIA | 5 mg/dl | 56.4 | 1.18 | 1.03 | 1.36 |
|  | | Chang-Yu Wang | 2009 | Taiwan | EC | 123 | NR | 97.6 | 17.9 | INA | 5 mg/L | 65.9 | 12.12 | 3.45 | 42.57 |
|  | | Urs Zingg | 2010 | Australia | EC | 90 | 63.6 | 82.2 | 83.3 | ITA | 10 mg/L | 22.2 | 5.07 | 1.92 | 13.43 |
|  | | Toshinobu Nakatsu | 2012 | Japan | ESCC | 73 | 64 | 79.5 | 41.1 | IHC | Stain score >= 6 | 58.9 | 6.61 | 1.98 | 22.02 |
| SCC-Ag | | Yoshihiro Nabeya | 2002 | Japan | ESCC | 29 | NR | NR | NR | EIA | 1.5ng/ml | 27.6 | 1.24 | 0.23 | 6.77 |
|  | | Hideaki Shimada | 2003 | Japan | ESCC | 215 | NR | NR | NR | EIA | 1.5ng/ml | NR | 1.87 | 1.19 | 2.95 |
|  | | Shin-ichi Kosugi | 2004 | Japan | ESCC | 245 | NR | 86.9 | 48.6 | ELISA | 1.5ng/ml | 30.6 | 0.92 | 0.58 | 1.45 |
|  | | Yutaka Shimada | 2005 | Japan | ESCC | 103 | 64.3 | 85.4 | 63.1 | EIA | 1.5ng/ml | 34 | 1.24 | 0.60 | 2.54 |
|  | | Mei Cao | 2009 | China | ESCC | 108 | 58.9 | 78.7 | 39.8 | RIA | 1.2ng/ml | NR | 1.10 | 0.43 | 2.86 |
| Hb | | D. Brattstrom | 2005 | Sweden | EC | 40 | NR | 72.5 | NR | NR | 12g/dl | 75.7 | 1.00 | 0.97 | 1.03 |
|  | | K.-L. Zhao | 2006 | China | ESCC | 303 | 57 | 69 | NR | NR | Female 11g/dl,male 12g/dl | 80.2 | 0.57 | 0.41 | 0.80 |
|  | | Javier Valencia Julve | 2006 | Spain | EC | 85 | 57.8 | 97.6 | 51.8 | NR | 13g/dl | 49.4 | 0.95 | 0.93 | 0.97 |
|  | | S. Zenda | 2008 | Japan | ESCC | 48 | 62.5 | 91.7 | 0 | NR | 13g/dl | 56.3 | 0.35 | 0.13 | 0.90 |
|  | | Dirk Rades | 2008 | Germany | EC | 68 | NR | NR | NR | NR | 12g/dl | 37 | 0.40 | 0.23 | 0.70 |

Abbreviations: HR, hazard ratio; ll, lower limit; ul, upper limit; IHC, immunohistochemistry; PCR, polymerase chain reaction; SSCP, single-strand conformation polymorphism; ELISA,enzyme-linked immunosorbent assay; LPIA, latex photometric immunoassay; LEHIA, latex-enhanced homogeneous immunoassay; INA, immunonephelometry; ITA, immunoturbidimetry; RIA, radioimmunoassay; EIA, enzyme immunoassay; NR, not report.
